# Antiangiogenic Tyrosine Kinase Inhibitors have Differential Efficacy in Clear Cell Renal Cell Carcinoma in Bone

**DOI:** 10.1158/2767-9764.CRC-24-0304

**Published:** 2024-10-08

**Authors:** Stefan Maksimovic, Nina C. Boscolo, Ludovica La Posta, Sergio Barrios, Mohammad Jad Moussa, Emanuela Gentile, Pedro I. Pesquera, Wenjiao Li, Jianfeng Chen, Javier A. Gomez, Akshay Basi, Jared K. Burks, Christopher Alvarez-Breckenridge, Jianjun Gao, Matthew T. Campbell, Eleonora Dondossola

**Affiliations:** 1 Department of Genitourinary Medical Oncology, David H. Koch Center for Applied Research of Genitourinary Cancers, The University of Texas MD Anderson Cancer Center, Houston, Texas.; 2 Division of Cancer Medicine, Department of Genitourinary Medical Oncology, The University of Texas MD Anderson Cancer Center, Houston, Texas.; 3 Department of Bioengineering, Rice University, Houston, Texas.; 4 Division of Surgery, Department of Urology, The University of Texas MD Anderson Cancer Center, Houston, Texas.; 5 Department of Leukemia, The University of Texas MD Anderson Cancer Center, Houston, Texas.; 6 Division of Surgery, Department of Neurosurgery, The University of Texas MD Anderson Cancer Center, Houston, Texas.

## Abstract

Clear cell renal cell carcinoma (ccRCC) is the most prevalent kidney neoplasm; bone metastasis (BM) develops in 35% to 40% of metastatic patients and results in substantial morbidity and mortality, as well as medical costs. A key feature of ccRCC is the loss of function of the von Hippel–Lindau protein, which enhances angiogenesis via vascular endothelial growth factor release. Consequently, antiangiogenic tyrosine kinase inhibitors (TKI) emerged as a treatment for ccRCC. However, limited data about their efficacy in BM is available, and no systematic comparisons have been performed. We developed mouse models of bone and lung ccRCC tumors and compared their anticancer efficacy, impact on mouse survival, and mechanisms of action, including effects on tumor cells and both immune and nonimmune (blood vessels and osteoclasts) bone stromal components. This approach elucidates the efficacy of TKIs in ccRCC bone tumors to support rational interrogation and development of therapies.

**Significance:**

TKIs showed different efficacy in synchronous bone and lung metastases and did not eradicate tumors as single agents but induced extensive reprogramming of the BM microenvironment. This resulted in a significant decrease in neoangiogenic blood vessels, bone remodeling, and immune cell infiltration (including CD8 T cells) with altered spatial distribution.

## Introduction

Clear cell renal cell carcinoma (ccRCC) represents 70% to 80% of renal neoplasms, with bone metastasis (BM) as a major manifestation of distant spread (35%–40% of patients; refs. 1–4). BM causes a variety of skeletal-related complications, including pain, spinal cord compression, hypercalcemia, mobility issues, and fractures (5, 6), thus posing a significant negative impact on patient quality of life and survival. Therapeutic resistance in BM confers significantly worse clinical outcomes, such as time-to-treatment failure, progression-free survival (PFS), and overall survival (OS; 7–9). In addition, BM generates substantial costs to society: it has been estimated that ∼20% of cancer-related expenditures are attributed to their management (7).

As a very distinctive feature of ccRCC, a driving mutation or epigenetic silencing of the tumor suppressor gene von Hippel–Lindau (VHL; ∼80–90% of the patients) leads to vascular endothelial growth factor (VEGF) release and increased angiogenesis (10–12). Consequently, small-molecule tyrosine kinase inhibitors (TKIs) targeting VEGF receptors (VEGFR) were developed for ccRCC treatment. Since 2006, seven antiangiogenic TKIs have been approved by the FDA in the United States, with axitinib, cabozantinib, and lenvatinib (13–15) currently prioritized in patients as first-, second-, or third-line treatments, alone or in combination with other treatments, mainly immune checkpoint inhibitors (ICI; refs. 16–20). However, no preclinical or clinical studies have directly compared the efficacy of different TKIs in bone metastatic tumors. Consequently, the optimal therapy for patients with bone metastatic ccRCC is yet to be defined (21). This assumes particular relevance due to the intrinsic target promiscuity of these agents that target several kinases other than VEGFR (22, 23), potentially enhancing efficacy and reducing drug resistance or increasing off-target toxicity (24). Additionally, despite the common use of TKIs in conjunction with ICIs, there is a lack of information about their impact on immune infiltration in BM. For these reasons, a thorough understanding of their mechanism of action and a systemic comparison of response (and resistance) within bone is urgently needed to provide treatment guidance and maximize the therapeutic benefit for patients with BM.

The aim of this study is to elucidate the efficacy of the most clinically used TKIs (axitinib, cabozantinib, and lenvatinib) in ccRCC in bone, including their impact on tumor, endothelial, immune, and bone cells. For this purpose, we developed experimental mouse models of bone tumors (based on UM-RC-3 and RENCA VHL^−^ cells, and for further confirmation studies, LVRCC67 cells) and performed *in vivo* treatment and macroscopic evaluation of survival after TKI treatment, as first- or second-line treatments, combined with microscopy-based spatial studies of response at the tissue, cellular, and subcellular levels.

## Materials and Methods

### Cells and reagents

Murine renal adenocarcinoma cell line RENCA (RRID: CVCL_2174) was obtained from ATCC; deletion of the VHL protein and lentiviral transduction with GFP and luciferase are described in the next paragraphs. The LVRCC67 cell line, generously provided by Dr. Ari Hakimi, was previously derived from a ccRCC mouse model (25). The UM-RC-3 (RRID: CVCL_2740) human primary renal adenocarcinoma cell line expressing GFP and luciferase was kindly provided by Dr. Katy Rezvani. The RENCA and UM-RC-3 cell lines were cultured in DMEM with 10% FBS and penicillin/streptomycin 5% at 37°C, 5% CO_2_. TKIs (axitinib, cabozantinib, and lenvatinib >99% purity) were purchased from TargetMol. The LVRCC67 cell line was cultured in K1 media prepared as described (25). Human vein umbilical cord cells (HUVEC) were from ATCC (CRL-4053). HUVECs (RRID:CVCL_2959) were cultured in endothelial cell growth base media (R&D Systems). The absence of *Mycoplasma* contamination was routinely verified. Cells were kept in culture for a maximum of 1 month.

### Deletion of VHL in the RENCA cell line

Vhl knockout (KO) in RENCA cells was generated with the CRISPR-Cas9 system. Briefly, crRNA targeting mouse Vhl (5′−CGT​TCC​AAT​AAT​GCC​CCG​GA−3′; Integrated DNA Technologies) in 1x Tris- ethylenediaminetetraacetic acid (EDTA) buffer was mixed with ATTO-550–labeled tracrRNA at equal concentrations and heated to 95°C for 5 minutes to form the crRNA–tracrRNA duplex. Duplexed sgRNA was then mixed with the Cas9 protein at a 1.2:1 ratio and incubated for 20 minutes at room temperature to form a ribonucleoprotein complex for transduction. Parental RENCA cells were trypsinized, rinsed twice with ice-cold PBS, and counted. Around 2 × 10^5^ cells were mixed with the sgRNA: Cas9 solution and electroporated with the Neon Transfection System (Thermo Fisher Scientific) by 1 pulse of 20 minutes at 1,800 V. Electroporated cells were then cultured for 1 day and sorted based on positive fluorescence from ATTO-550 in 96-well plates for isolating single clones. Successfully isolated single KO clones were verified by Western blotting and Sanger sequencing analysis of an amplicon around the expected cut site.

### Western blot

RENCA (wild-type) and RENCA VHL^−^ were lysed using RIPA buffer (containing 50 mmol/L Tris, 150 mmol/L NaCl, 1 mmol/L EDTA, 1% IGEPAL, and 1% glycerol), supplemented with Complete Protease Inhibitor Cocktail (Roche), PhosSTOP Phosphatase Inhibitor Cocktail (Roche), 100 mmol/L vanadate (Invitrogen Life Technologies), and 1 mmol/L dithiothreitol (Sigma-Aldrich). Protein concentration was determined with Bradford assay (Bio-Rad). Proteins were separated in 10% to 12% Bis-Tris gels and transferred to polyvinylidene difluoride membranes using the Trans-Blot Turbo Transfer System. The membrane was incubated with a primary antibody for VHL (dilution 1:1,000; sc-17780 Santa Cruz Biotechnology) overnight at 4°C. Horseradish peroxidase–conjugated antibodies in 1:1,000 dilution were used as secondary antibodies for 1 hour at room temperature. The Image Manager system was used for proteins detection and analysis (KwikQuant, Kindle Biosciences, LLC).

### Generation of GFP and luciferase variants of RENCA VHL^−^

To generate GFP- and luciferase-positive cells, RENCA VHL^−^ cells were stably transduced with rLV.EF1.mGFP lentiviral vector, luciferase lentiviral vectors (Vectalys; 1 × 10^6^ TU/10^6^ cells), and 0.5 μL of polybrene in 1 mL of DMEM complete medium in a 24-well plate (Thermo Fisher Scientific) overnight and expanded.

### Cell viability and proliferation assays

UM-RC-3, RENCA VHL^−^, and HUVECs were seeded in a 96-well plate (*n* = 5 wells/group/dose, 2,000 cells/well cancer cells; *n* = 4 wells/group/dose, 10,000 cells/well HUVECs). After 24 hours, TKIs were diluted in maximum 1% DMSO (used as a control) in complete cell culture medium (10 pmol/L–1 mmol/L), and treatments were applied for 72 hours. CellTiter-Glo Luminescent Cell Viability Assay was utilized to assess the viability of tumor cells, followed by quantification of the bioluminescence signal. HUVECs were imaged after 72 hours using the EVOS FL Cell Imaging System (AMG) equipped with 4× and 10× objectives, and the GFP area occupied by the cells was analyzed using Image J (26).

### Animal studies

Animal studies were approved by the Institutional Animal Care and Use Committee of The University of Texas MD Anderson Cancer Center and performed according to the institutional guidelines for animal care and handling. All the procedures described were performed in agreement with the NIH Policy on Humane Care and Use of Laboratory Animals. Eight-week-old female C57/Bl6 (RRID: MGI:7264769), BALB/c (RRID: MGI:2683685), and NOD.Cg-Prkdc^scid^/J (SCID; RRID: IMSR_JAX:001303) mice were obtained from The Jackson Laboratory. The mice were housed with a maximum of five animals per cage in a state-of-the-art, air-conditioned, specific pathogen–free animal facility. Surgeries were performed with mice under general anesthesia (isoflurane), and analgesia was provided for each procedure (buprenorphine slow release, 0.5 mg/kg, immediately before the start of the surgery). Tumor-bearing animals were observed daily and examined by a veterinarian 5 days/week for signs of morbidity (e.g., matted fur, weight loss, limited ambulation, and respiratory difficulty). In case of discomfort, the animals were euthanized by asphyxiation with carbon dioxide gas followed by cervical dislocation, consistent with the recommendations of the Panel on Euthanasia of the American Veterinary Medical Association.

### *In vivo* tumor inoculation and treatment

BALB/c mice (*n* = 8–10 mice/group) were injected in both tibiae with 2 × 10^5^ VHL^−^ GFP^+^ luc^+^ RENCA cells on day 0. NOD.Cg-Prkdc^scid^/J mice (*n* = 8–10 mice/group) were injected in both tibiae with 1 × 10^6^ GFP^+^ luc^+^ UMRC-3. Tumor growth was detected via bioluminescence detection after retro-orbital administration of luciferin (3.75 mg/mL in PBS immediately before imaging). The mice were randomized on day 7 (RENCA VHL^−^) or 21 (UM-RC-3) after tumor cell injection based on the macroscopic bioluminescence signal in the tibia and treated with vehicle (7% DMSO, 30% polyethylene glycol 300, and 5% TWEEN 10% v/v), cabozantinib 40 mg/kg/day, lenvatinib 30 mg/kg/day, or axitinib 25 mg/kg/day, for up to 70 days, depending on the experimental schedule. C57/Bl6 mice (*n* = 5 mice/group) were injected in both tibiae with 3 × 10^5^ LVRCC67 cells on day 0 and treated with TKIs after 14 days for 6 days by oral gavage, as described above.

### Tissue processing, immunofluorescence, and imaging

At different experimental time points, the mice were sacrificed, and bones and lungs were collected. Tissue was fixed in 4% paraformaldehyde overnight, and bones were further decalcified in 0.5 mol/L EDTA for 5 to 7 days. Bones and lungs were embedded in 4% and 10% agarose, respectively, and sliced using a Leica vibratome, generating 250-μm-thick and 100-μm-thick slices, respectively. The slices were blocked overnight at 4°C in a diluent (10% DMSO, 5% normal donkey serum, and 0.5% IGEPAL in PBS). Then, the slices were incubated with the following primary antibodies: endomucin (sc-65495, Santa Cruz Biotechnology; RRID: AB_2100037), laminin 1 to 2 (ab7463, Abcam, RRID: AB_305933), CD31 (AF3628, R&D Systems, RRID: AB_2161028), CD8 (14-0195-82, Invitrogen, RRID: AB_2637159), tartrate-resistant acid phosphatase (AB185716, Abcam, RRID:AB_3095617), and PAX8 (AB 239363, Abcam) in a diluent at 4°C overnight. The slices were washed 3 to 5 times in cold PBS for 10 minutes and further incubated with secondary antibodies (1:400 in a diluent) conjugated with Alexa Fluor dye 488- 750 for 2 hours. Following incubation with secondary antibodies, the slices were washed 5 times in cold PBS and incubated in 4′,6-diamidino-2-phenylindole (DAPI) solution (1:2,000, in diluent) for 10 minutes at 4°C. Finally, the slices were washed 3 times in PBS. Immunofluorescence (IF) images were captured using an SP8 Leica confocal microscope equipped with 40× and 100× water and oil immersive objectives, respectively, without utilizing optical zoom. Images were captured at a 2.9 px/μm resolution, and a mosaic was generated based on the size of the region of interest. 3D stacks had 7 μm step intervals in z-direction obtained for total depth between 28 and 56 μm. The acquired images and mosaic were processed/merged initially by LasX software from Leica. Macroscopic images of the lungs *ex vivo* before processing were captured using a Leica stereomicroscope in brightfield and with an epifluorescence lamp with excitation at 488 nm.

### Digital image processing and analyses

Images were reconstructed, stitched, and analyzed using Image J [(unless specified differently) RRID: SCR_003070; ref. 26].

#### Area analysis

All quantitative analyses were performed on maximum projection of 3D stacks, except in lungs in which single slices were analyzed separately. Single channels were masked, thresholded (default), and converted to binary images; the signal-positive area was obtained and reported as percentage of the total area analyzed (e.g., the area of tumor blood vessels related to the area occupied by tumor cells). For each slice of lungs, the relative fluorescence density was obtained from 4 to 6 slices per z-stack, averaged, and represented as the percentage of the total area. Up to two representative slices/tumor were used, 3 to 5 tumors/group.

#### Reciprocal distance of blood vessels in the tumor area

Manual analysis of the minimum distance between two closest vessels was performed on 10 to 20 vessels randomly selected in the tumor area.

#### Quantification of CD8^+^ cells

The number of CD8^+^ cells associated with tumor in bone and lungs was manually quantified for each time point using the Cell Counter plugin (Kurt De Vos University of Sheffield, Academic Neurology) of ImageJ (NIH). To quantify the distribution of CD8^+^ associated with tumor, the tumor margin was defined based on the GFP signal expressed by cells and included the area ±25 μm distant from the tumor/bone marrow interface. The distribution of CD8^+^ was expressed as the percentage/tumor area.

#### Distance of CD8^+^ cells from the closest blood vessels

Approximately 30 random cells for up to six slices were chosen, and the distance was calculated manually as the shortest straight line that connected the cell to the closest blood vessel and distance measured.

### COMET multiplex analysis

Bones were fixed in 4% paraformaldehyde overnight and further decalcified in 0.5 mol/L EDTA for 5 to 7 days. Bones were paraffin-embedded and sliced on a Leica microtome (8 μm thickness). The slices were further deparaffinized, and antigen retrieval was performed in basic EDTA (pH 9, 0.1 mol/L) at 107°C for 15 minutes in the EZ-Retriever Microwave System (BioGenex). The slides were quenched for autofluorescence in 10% hydrogen peroxide for 10 minutes. Eight-μm-thick slices were placed on the slides to fit an 8 × 8 mm chip and further acquired using Lunaphore Comet PA at MD Anderson’s Flow Cytometry and Cellular Imaging Facility. Markers of interest were detected using the following antibodies: Ly6C (Bio-Rad, MCA2389GA, RRID: AB_844551), CD11c (Cell Signaling Technology—39143, RRID: AB_2924836), CD3e (CST, 73484), B220 (Thermo Fisher Scientific, 14-0452-82, RRID: AB_467254), CD4 (Thermo Fisher Scientific, 14-9766-82, RRID: AB_2573008), CD8 (Thermo Fisher Scientific, 14-0195-82, RRID: AB_2637159), FoxP3 (Thermo Fisher Scientific, 14-5773-82, RRID: AB_467576), CD11b (Proteintech, 21851-1-Ap, RRID: AB_2878927), Ly6G (Proteintech, 65140-1-Ig, RRID: AB_2881475), and F480 (Proteintech, 28463-1-Ap, RRID: AB_2881149). All acquired images were first processed for background subtraction in COMET viewer software provided by Lunaphore by measuring the autofluorescence in the tissue from an unstained initial cycle. Image analyses were performed in Vis Software (Visiopharm); this process included tissue detection and segmentation algorithms (Decision Forest) and a deep learning algorithm for cell segmentation (UNET), followed by cell phenotyping and quantification by using different modalities in the same software, including machine learning, deep machine learning, and artificial intelligence. The absolute number of cells defined by phenotyping was expressed over the tumor area. The same criteria applied to define the tumor interface mentioned above were utilized to quantify cell distribution.

### Micro-CT

Changes in bone volume and bone microarchitecture in tibiae (*n* = 4/group) were quantified *ex vivo* using a SkyScan 1276 Micro-CT (μCT) scanner (Bruker). Tibiae were placed individually in 1.5-mL microtubes filled with PBS and scanned at a voxel resolution of 13 μm. 3D μCT scans were reconstructed using NRecon (Bruker), and data analysis was completed using CTan (Bruker) in which data were separated into two separate regions, the metaphyseal trabecular bone and the diaphyseal cortical bone. μCT measurements included bone volume fraction, trabecular number, trabecular separation, trabecular thickness, cortical bone thickness, and bone surface density.

### Statistical analysis

Statistical analysis was performed using GraphPad Prism 9.0 (GraphPad Software; RRID: SCR_002798). To test differences between two populations, the unpaired two-sided Student *t* test was applied. To test the differences among more than two populations, one-way ANOVA was performed, followed by Tukey honestly significant difference *post hoc* test. For the survival (Kaplan–Meier) curve analysis, the log-rank (Mantel–Cox) test was performed. All statistical tests were two-sided, and a *P* value of less than 0.05 was considered statistically significant.

### Data availability

The original data presented in this study are included in the article and Supplementary Material. The raw and analyzed datasets generated during the study, too large to be publicly shared, are available from the corresponding author upon reasonable request.

## Results

### Development of ccRCC models in bone

**Figure 1:**
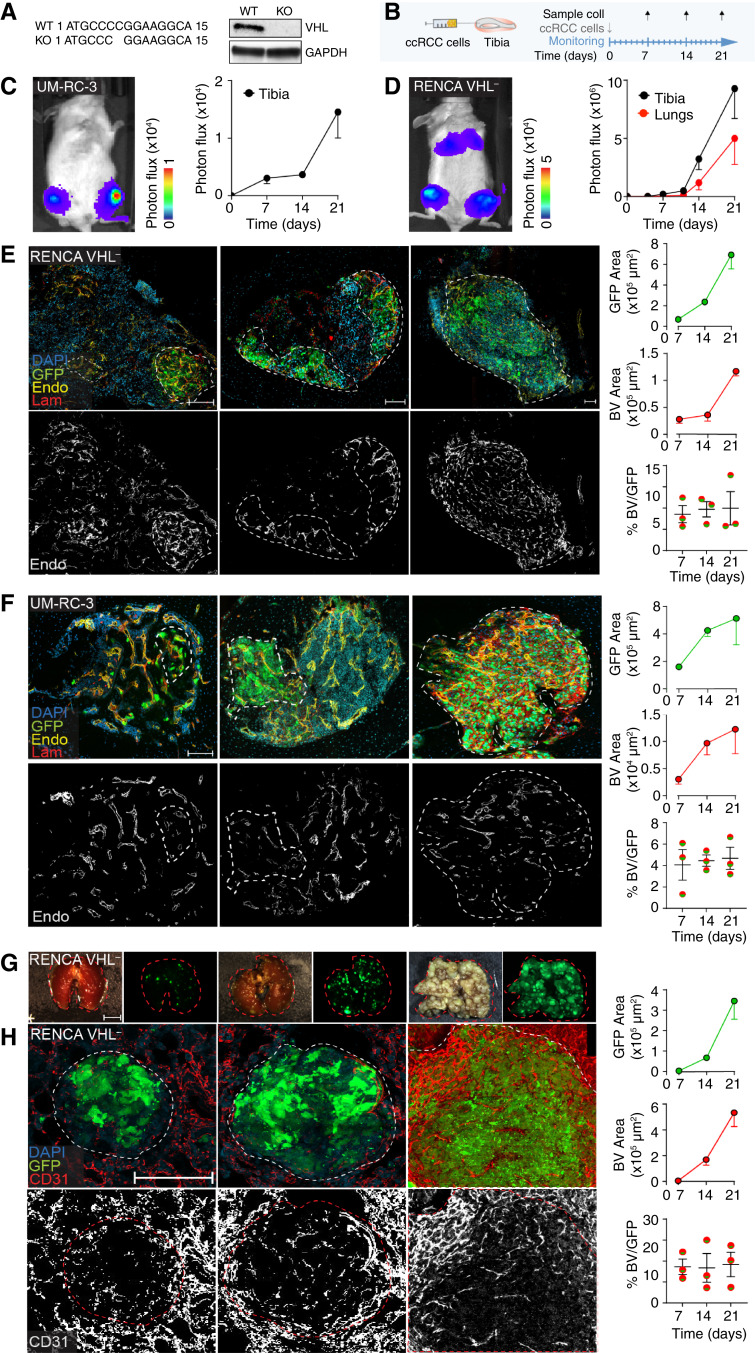
Development of ccRCC models in bone. **A,** WT and KO sequence of the *vhl* gene of RENCA cells and Western blot analysis of VHL protein expression compared with the housekeeping protein, GAPDH. **B,** Schematic representation of the experimental models. Tumor cells were implanted into the tibia, and tissues were collected 7, 14, and 21 days after implantation. **C** and **D,** Tumor progression [UM-RC-3 (**C**) and RENCA VHL^−^ (**D**) monitored *in vivo* by bioluminescence, mean ± SEM, *n* = 8–10 tibiae/group]. **E** and **F,** Confocal microscopy analysis of tumors in tibiae (RENCA VHL^−^, **E**; UM-RC-3, **F**). A quantification of tumor (green, GFP) and blood vessel (endomucin, yellow; laminin, red) areas over time is shown. Nuclei (blue, DAPI). Bar, 100 µm, mean ± SEM, *n* = 8–10 tibiae/group. **G,** Stereomicroscope images of lungs (brightfield and GFP); bar, 0.5 cm. **H,** Confocal microscopy analysis of tumors in lungs (RENCA VHL^−^); a quantification of tumor (green, GFP area) and blood vessel (CD31, red) areas over time is shown. Nuclei (blue, DAPI). Bar, 100 µm. Mean ± SEM, *n* = 8–10 tibiae/group. BV, blood vessel; Endo, endomucin; Lam, laminin; WT, wild-type.

To investigate the impact of different TKI agents in ccRCC bone lesions, we first established and characterized RENCA murine and UM-RC-3 human cell line growth in bone. To recapitulate key features of ccRCC progression in patients, VHL^+^ RENCA cells were genetically modified by CRISPR-Cas9 to KO the *vhl* gene (27, 28). The absence of protein expression was confirmed by Western blot analysis (Fig. 1A). UM-RC-3 cells, instead, already have an intrinsic inactivating missense *vhl* mutation (L89H), also identified in patients with ccRCC (29, 30).

Bone tumors were generated by direct implantation of cancer cells in mouse tibia, an established approach that supports consistency and reproducibility for testing therapeutic agents (31–36). ccRCC cells, engineered to express GFP and luciferase (which allows quantitative and noninvasive longitudinal monitoring of tumor progression), were injected into tibiae (RENCA VHL^−^, 2 × 10^5^ cells/tibia; UM-RC-3, 1 × 10^6^ cells/tibia), and their growth was detected by bioluminescence analyses (Fig. 1B–D). Interestingly, the intratibial injection of RENCA VHL^−^ cells led to further lung colonization, most likely via intravasation into the venous systems, allowing a comparison of tumor progression in two different metastatic sites (Fig. 1D). However, lung colonies were not observed in mice implanted with UM-RC-3 in bone. ccRCC lesions were detected as early as day 5 after injection, and tumor growth in tibiae and lungs (for RENCA VHL^−^) was exponential (Fig. 1C). RENCA VHL^−^ did not show evidence of rejection due to GFP and luciferase expressions in either bones or lungs (Fig. 1D).

To monitor tumor growth progression at the cellular level in both models, tibiae and lungs were collected at days 7, 14, and 21 post-injection and analyzed by IF. This analysis confirmed that tumor cells, visualized by GFP expression, progressively colonized the bone cavity and replaced bone marrow at later time points in both RENCA VHL^−^ and UM-RC-3 models (Fig. 1E and F). Blood vessels in bone were detected by laminin staining, expressed in the basal membranes of every blood vessel, and endomucin, which is expressed in sinusoidal blood vessels (Supplementary Fig. S1A). Interestingly, neovessels displayed both markers with no significant differences, showing that neoangiogenic tumor vasculature in bone retains molecular characteristics of sinusoidal blood vessels (Supplementary Fig. S1B–S1D). Additionally, progression of RENCA VHL^−^ cell colonies in the lungs led to the near-complete replacement of the alveolar space (Fig. 1G and H), a lethal event for the mouse (and a limiting step for further follow-up of bone lesion progression). Blood vessels in lungs were visualized via CD31 expression. Blood vessel network development paralleled tumor progression, maintaining a constant density within the tumor area over time in both models and metastatic sites (Fig. 1D and E). As ccRCC BM are majorly osteolytic, we monitored the presence of this feature in our models. Both RENCA VHL^−^ and UM-RC-3 tumor cells induced activation of osteoclasts and consequent bone resorption, in line with the phenotype identified in patients with ccRCC BM (Supplementary Fig. S2A and S2B; ref. 37).

As a result, we established and characterized models of ccRCC progression in bone and lungs.

### Efficacy of TKIs on tumor growth, *in vitro* and *in vivo*

**Figure 2:**
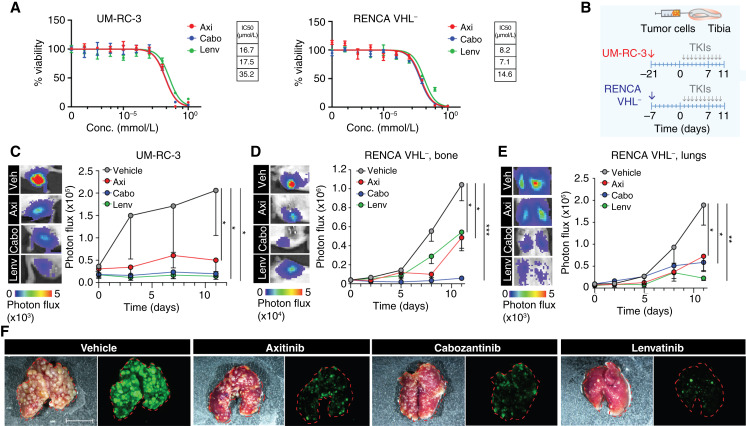
Efficacy of TKIs on tumor growth, *in vitro* and *in vivo*. **A,** Dose–response curve of axitinib, cabozantinib, and lenvatinib treatment of UM-RC-3 and RENCA VHL^−^, mean + SD, *n* = 5/group; control, 0 mmol/L TKIs, 1% DMSO; IC_50_ values of cell viability are reported. **B,** Experimental design and timeline of treatment schedule. **C** and **D,** Response to TKIs by UM-RC-3 (**C**) and RENCA VHL^−^ (**D**) tumor cells in tibiae at day 11 of treatment; representative bioluminescence images and quantification are shown, mean ± SEM, *n* = 16 tibiae/group. **E,** Response of tumor cells in lungs to TKIs at day 11 of treatment; representative bioluminescence images and quantification are shown, mean ± SEM, *n* = 8/group. **F,** Stereomicroscope images of lungs (brightfield and GFP–green); Bar, 1 cm. *P* values by one-way ANOVA with Tukey honestly significant difference *post hoc* test; *, *P* < 0.05; **, *P* < 0.01; ***, *P* < 0.001. Axi, axitinib; Cabo, cabozantinib; Conc, concentration; Lenv, lenvatinib; Veh, vehicle.

As the next step, we compared the efficacy of antiangiogenic TKIs in our models. We focused on axitinib, cabozantinib, and lenvatinib, the three TKIs currently prioritized for treating patients with ccRCC (21). These TKIs target VEGFR and multiple other kinases [such as PDGFβ, c-MET, RET, AXL, FLT3, Tie2 FLT3, Tie2, and FGFR1 (38–49)]. First, we tested the cytotoxicity of TKIs on RENCA VHL^−^ and UM-RC-3 tumor cells after 72 hours of incubation *in vitro*. The TKIs significantly impaired cellular viability with the following IC_50_ values in UM-RC-3 and RENCA VHL^−^, respectively: axitinib—16.7 μmol/L and 8.2 μmol/L; cabozantinib—17.5 μmol/L and 7.1 μmol/L; and lenvatinib—35.2 μmol/L and 14.6 μmol/L; Fig. 2A, in line with published results across different cancer types (38–49). Then, we compared the outcome of TKIs on tumor growth *in vivo*. Balb/c and NOD-SCID mice were injected with luciferase- and GFP-expressing RENCA VHL^−^ and UM-RC-3 cells, respectively, and randomized 7 or 21 days post-injection. Because axitinib, lenvatinib, and cabozantinib are administered orally to patients at different doses (13–20), we selected among effective doses commonly applied in preclinical experiments (31, 42, 50–53) that reflected clinically relevant ones and administered 25, 30, and 40 mg/kg, respectively, orally, daily, until the end of the experiment (day 10 post-treatment; Fig. 2B). This treatment scheme was further applied in the *in vivo* experiments described in this work (unless a prolonged treatment time was indicated).

All three drugs significantly inhibited the growth of UM-RC-3 and RENCA VHL^−^ cells in bone, with cabozantinib showing relatively higher efficacy compared with lenvatinib and axitinib in RENCA VHL^−^ lesions but not in UM-RC-3 tumors (Fig. 2C and D). Due to the lung seeding, we further assessed the efficacy of TKIs in RENCA VHL^−^ lung lesions, in which they all showed similar efficacy compared with control-treated mice (Fig. 2E and F).

To assess the potential impact of initial tumor size on the activity of TKIs, we performed a follow-up experiment on more established tumors that grew for 14 days before treatment (reaching a four-fold lesion size in bone based on the bioluminescence signal at treatment initiation). All TKIs showed identical efficacy, suggesting that the initial tumor size does not impair their activity (Supplementary Fig. S3A).

These results suggest that TKIs significantly reduce tumor progression while not eradicating bone or lung tumors up to 10 days post-treatment, as expected for antiangiogenic agents. In addition, TKIs demonstrated efficacy in an immunodeficient mouse model of ccRCC in bone, suggesting that therapeutic response mechanisms are independent of the adaptive immune system.

### TKIs inhibit neoangiogenesis in bone and lung tumors

**Figure 3:**
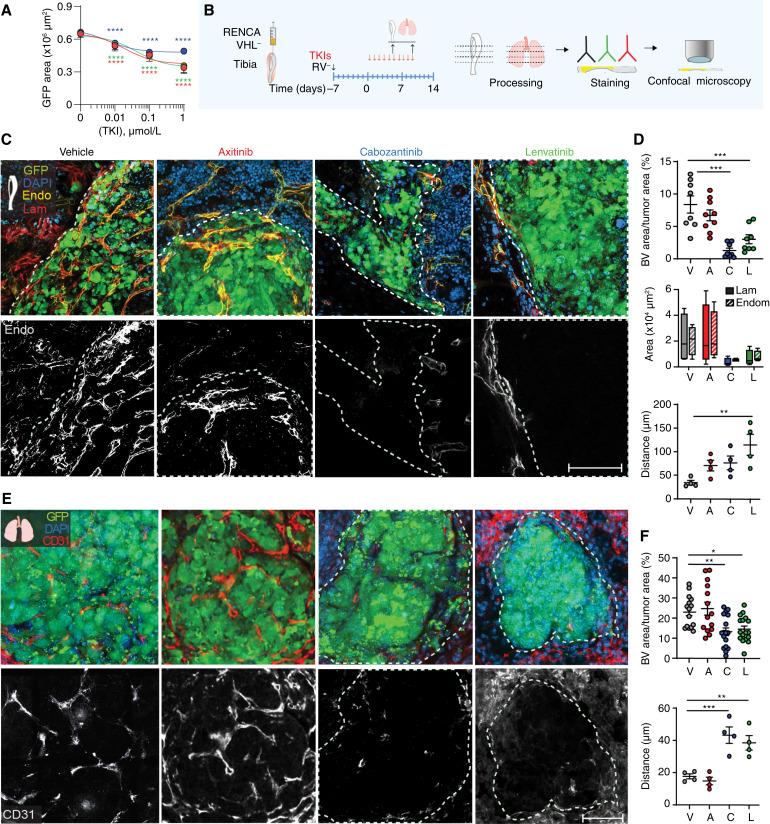
*In vitro* and *in vivo* effects of TKIs on blood vessels. **A,** Dose–response curve of axitinib, cabozantinib, and lenvatinib treatment of HUVECs, mean ± SD, *n* = 4. **B,** Schematic representation of the experimental approach. **C** and **D,** Impact of TKIs on vascularization of RENCA VHL^−^ tumors in bone evaluated by IF analysis; representative images acquired by confocal microscopy (green, GFP; blue, DAPI; yellow, endomucin; red, laminin; **C**) and quantification are shown (**D**); dotted line, tumor area; mean ± SEM, *n* = 4–10/group; bar, 100 μm. **E** and **F,** Impact of TKIs on vascularization of RENCA VHL^−^ tumors in lungs; representative images acquired by confocal microscopy (green, GFP; blue, DAPI; red, CD31; **E**) and quantifications are shown; dotted line, tumor area; mean ± SEM, *n* = 4–10/group; ar, 100 μm. *P* values by one-way ANOVA with Tukey honestly significant difference *post hoc* test; *, *P* < 0.05; **, *P* < 0.01; ***, *P* < 0.001. A, axitinib; BV, blood vessel; C, cabozantinib; Endo/Endom, endomucin; L, lenvatinib; Lam, laminin; RV^−^, RENCA VHL; V, vehicle.

Next, we investigated the impact of TKIs on blood vessel biology. To assess the impact of TKIs on endothelial cells *in vitro*, we treated human endothelial cells expressing GFP and quantified cell growth. All three drugs significantly decreased the proliferation of HUVECs at 10 nmol/L (Fig. 3A), a suboptimal concentration to impact tumoral cell growth (Fig. 2A). To elucidate mechanisms of action and efficacy of TKIs on angiogenesis *ex vivo* at the cellular level, we performed IF analysis of blood vessels in tumors. Mice treated with TKIs for 6 or 10 days were sacrificed, and IF analyses of endomucin and laminin expressions in bone and CD31 in lungs (Fig. 3B–E; Supplementary Figs. S4 and S5) were performed. We identified a significantly reduced amount of blood vessels in bone tumors treated with TKIs at day 6 post-treatment, whereas axitinib did not significantly decrease blood vessel formation in lung lesions (Supplementary Fig. S4A–S4E). Noticeably, cabozantinib and lenvatinib halted blood vessel formation in bone tumors up to day 10 post-treatment, whereas axitinib-treated animals showed a higher number of blood vessels at this time point (Fig. 3C–F; Supplementary Fig. S5).

Furthermore, endomucin and laminin expression levels showed no significant differences in tumor blood vessels of mice treated with TKIs versus control (Fig. 3D), suggesting that blood vessels maintained their original characteristics after treatment. Additionally, we measured the blood vessel reciprocal distance as an index of blood vessel density (Supplementary Fig. S6). Interestingly, lenvatinib-treated tumors showed significantly increased distance between proximal vessels, with most of them confined at the tumor–bone marrow interface. We confirmed our findings in UM-RC-3 tumors in bone (Supplementary Fig. S7). Likewise, we did not identify significant difference between laminin and endomucin expressions in neovessels. Furthermore, only cabozantinib and lenvatinib significantly decreased blood vessel density in tumor lesions after 10 doses, and the reciprocal distance between neovessels within tumors was significantly increased after TKI treatment (Supplementary Fig. S7).

In conclusion, we reported that the three TKI therapies can quantitatively change neovessels in bone and lung tumors differentially.

### TKIs significantly reduced immune infiltration in bone and lung tumors

The three TKIs used in this preclinical study are often used in combination with ICIs, which target PD-1 on CD8^+^ T cells and other immune cells (16–20). To further investigate the effects of TKIs on the tumor-associated immune microenvironment, we focused on RENCA VHL^−^ cell–implanted mice and confirmed the results in an additional immunocompetent mouse model of ccRCC in bone (LVRCC67).

**Figure 4:**
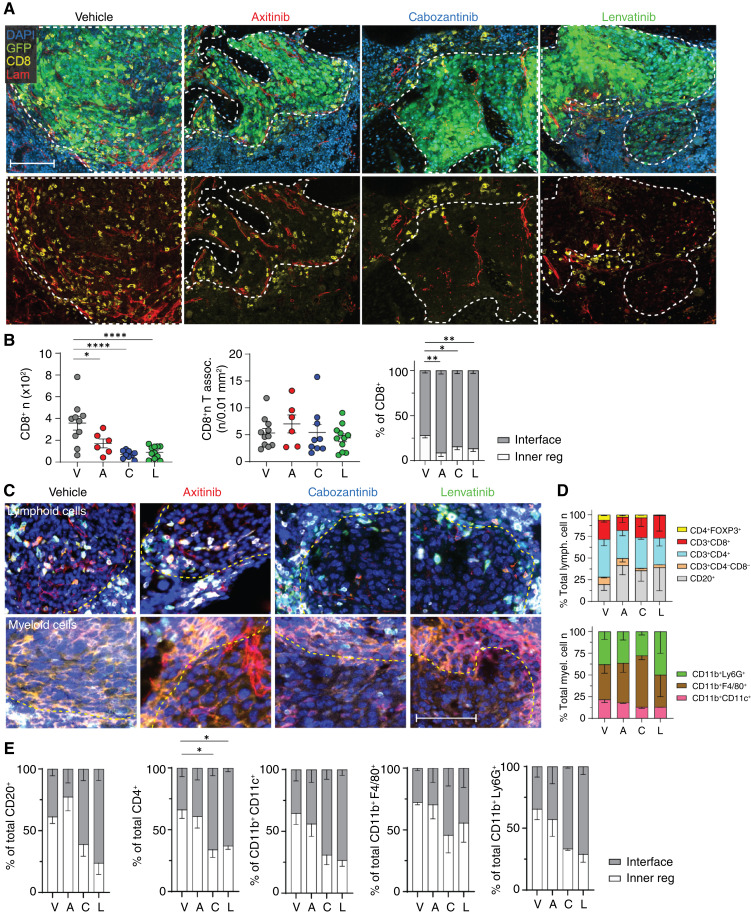
TKIs’ effect on immune cell infiltration in bone tumors. **A,** Impact of TKIs on CD8 infiltration in RENCA VHL^−^ tumors in bone evaluated by IF analysis; representative images acquired at the confocal microscope (green, GFP; blue, DAPI; yellow, CD8; red, laminin) are shown; dotted line, tumor area. Bar, 100 μm. **B,** Quantification of the number and distribution of CD8^+^ cells associated with tumor; mean ± SEM, *n* = 6–12/group. **C,** Representative images and quantification (**D** and **E**) of immune subsets infiltrating bone tumors by multiplex imaging (COMET, Lunaphore) post-TKI treatment, mean ± SEM, *n* = 3/group *P* values by one-way ANOVA with Tukey honestly significant difference *post hoc* test; *, *P* < 0.05; **, *P* < 0.01; ***, *P* < 0.0001. Inner tumor region >25 μm from the tumor edge. A, axitinib; C, cabozantinib; Inner reg, inner region; L, lenvatinib; Lam, laminin; lymph, lymphocytes; myel, myeloid; V, vehicle.

To address the impact of different TKIs on CD8^+^ T cells in ccRCC bone tumors, we focused on IF-based analysis, as opposed to other bulk or single-cell techniques that require tissue dissociation (e.g., PCR, flow cytometry, etc.) because of the consequent loss of spatial information associated with those analyses. This information is particularly relevant in the case of TKIs that directly target the tumor blood vessels in the microenvironment and, as a consequence, might impact the extravasation of immune cells into the tumor, influencing not only their number but also their distribution. First, we characterized their infiltration in RENCA VHL^−^ lesions over time (Supplementary Fig. S8A). The number of CD8^+^ T cells in treatment-naïve tumors gradually increased over time, showing an abundant infiltrate by day 21 post-implantation (Supplementary Fig. S8A), in line with published evidence of high T-cell infiltration in ccRCC (54, 55). To better understand the spatial distribution of CD8^+^ T cells within tumors, we defined two areas: the interface with the bone marrow (±25 μm from the tumor edge) and the inner tumor region (>25 μm from the tumor edge). Interestingly, the majority of CD8^+^ T cells remained at the interface between the bone marrow and tumor at days 7 and 14 post-implantation, yet more than 60% were intratumoral at day 21 (Supplementary Fig. S8A–S8C). Then, we investigated the impact of TKIs on the patterns of this infiltration (Fig. 4A and B). All TKIs significantly reduced the total number of CD8^+^ T cells associated with tumors (Fig. 4B). Because the tumor size is also reduced by TKIs, we calculated the number of CD8^+^ cells/tumor area in mm^2^, which remained constant across different groups, suggesting a constant average density of CD8^+^ cells in TKI-treated mice. However, their spatial distribution was impacted by treatment, with more than 70% of these lymphocytes located at the tumor–bone marrow interface (Fig. 4A and B). Axitinib-treated mice showed a trend toward an increased number of total CD8^+^ T cells, in line with incipient revascularization shown in Fig. 3C and D. In addition, the distance between blood vessels and CD8^+^ cells was not significantly affected by TKI treatment (Supplementary Fig. S8E and S8F), with lymphocytes located at 10 to 50 μm from the closest blood vessel, similar to control-treated bone tumors.

In lung lesions, CD8^+^ T lymphocytes in treatment-naïve tumors followed a similar trend (Supplementary Fig. S9A and S9B). Cabozantinib and lenvatinib significantly lowered the total number of CD8^+^ T cells infiltrating the tumor, with a predominant distribution of these cells at the tumor interface (Supplementary Fig. S9C and S9D), as shown in bone lesions. As previously mentioned, axitinib-treated lung tumors did not affect neoangiogenesis in lungs after both 6 and 10 doses, which resulted in a higher infiltration of CD8^+^ T cells comparable with untreated mice (Supplementary Fig. S9C and S9D). To further interrogate the impact of TKIs on overall immune infiltrating cells, we performed multiparametric sequential IF–based spatial analysis of RENCA VHL^−^–bearing bones after treatment. We detected eight different populations of myeloid and lymphoid cells (Fig. 4C–E), quantified their proportions, and defined their spatial distribution, as previously described for CD8^+^ T cells. Interestingly, among lymphoid subpopulations, CD3^+^CD4^+^ T cells and CD20^+^ cells were the most abundant, representing 25% to 45% of the immune infiltrate, followed by CD3^+^CD8^+^ T cells. The proportions of these cell subsets were not significantly affected by TKI treatment. The majority of immune subsets remained confined at the tumor–bone marrow interface after cabozantinib or lenvatinib treatment, whereas vehicle- and axitinib-treated tumors showed more infiltration. CD3^+^CD4^+^ T cells had the same pattern of infiltration of CD3^+^CD8^+^ T cells, likely driven by a marked reduction of blood vessels in cabozantinib- and lenvatinib-treated tumors (Fig. 4D). Interestingly, TKIs showed a trend toward increasing the number of CD20^+^ cells but decreasing the CD4^+^FoxP3^+^ (T-regulatory cells) population. Additionally, we identified and characterized three subpopulations of myeloid cells infiltrating the tumor in bone: CD11b^+^CD11c^+^, CD11b^+^Ly6G^+^, and CD11b^+^F4/80^+^, representing dendritic cells, neutrophils, and macrophages, respectively. Macrophages represented the most abundant subpopulation infiltrating these tumors, and this population was slightly increased in cabozantinib-treated tumors. However, the pattern of infiltration for all three lineages of CD11b^+^ was dependent on blood vessel presence, with cabozantinib- and lenvatinib-treated tumors showing a spatial pattern of infiltration similar to CD4^+^ and CD8^+^ cells (Fig. 4D). Overall, all tracked lymphoid and myeloid cell populations exhibited a tendency to infiltrate tumors contingent upon blood vessel abundance, with cabozantinib and lenvatinib showing a prominent confinement of immune cells along the tumor margin.

To confirm TKIs’ effect on blood vessel formation and the consequent limitation of the immune infiltrate, we developed an additional immunocompetent mouse model of bone lesions based on LVRCC67 cell injection. This ccRCC cell line was established from a novel electroporation-derived ccRCC syngeneic model that carries CRISPR-mediated deletion of *Vhl*, *Tp53*, and *Rb1* (25). LVRCC67 cells were injected in the tibia of C57/B6 mice; after 2 weeks, mice were administered 6 doses of TKIs via oral gavage, and mouse bones were retrieved for end-point evaluation of blood vessel and CD8^+^ cell infiltration by IF analyses. Because these tumor cells did not express GFP, an antibody against PAX8 (a nephric-lineage transcription factor expressed by kidney cells; ref. 56) was used to visualize them. Interestingly, TKI-treated mice showed an impact on blood vessels, consistent with our other two models (Supplementary Fig. S10A and S10B). Axitinib did not induce a significant reduction of the blood vessel area in tumors. However, cabozantinib and lenvatinib markedly reduced tumor vasculature, with more than 60% of CD8^+^ cells remaining at the tumor margin (Supplementary Fig. S10B). Furthermore, the total number of CD8^+^ cells per area of tumor was not changed compared with the control treatment, as we previously observed in our Balb/c model.

Altogether, these data suggest that the CD8^+^ cell phenotype induced by TKIs in tumors is consistent in different mouse models.

### Cabozantinib and lenvatinib alleviate osteolysis

Osteolytic lesions in ccRCC BM result in a high risk of skeletal-related events (37). To assess the effects of TKIs on osteolytic lesions, we performed an *ex vivo* μCT analysis on tibiae implanted with RENCA VHL^−^ cells and treated with 10 doses of TKIs.

**Figure 5:**
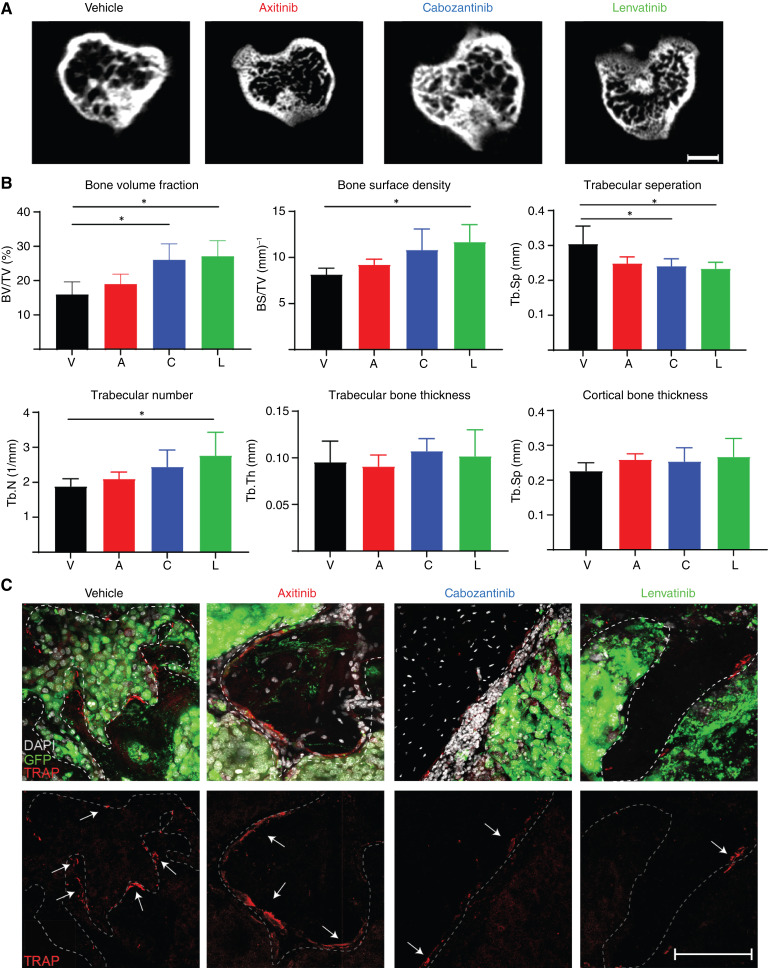
*Ex vivo* µCT analysis of TKIs’ effects on bone microarchitecture. **A,** Representative μCT micrographs of tibial metaphysis cross-sections extracted from mice treated with axitinib, cabozantinib, and lenvatinib or vehicle, Bar, 300 μm. **B,** Quantification of key bone histomorphometry parameters extrapolated from μCT analysis, mean ± SEM, *n* = 4/group. **C,** Impact of TKIs on TRAP^+^ cells in RENCA VHL^−^ tumors in bone evaluated by IF analysis; representative images acquired at the confocal microscope (green, GFP; white, DAPI; red, TRAP) are shown; arrow, TRAP^+^ cells. Bar, 250 μm. *P* values by one-way ANOVA with Tukey honestly significant difference *post hoc* test; *, *P* < 0.05. A, axitinib; C, cabozantinib; L, lenvatinib; TRAP, tartrate-resistant acid phosphatase; V, vehicle.

Bone analyses demonstrated that cabozantinib and lenvatinib significantly impacted bone resorption, especially in trabecular bone, with a significantly preserved trabecular number and reduced separation. Moreover, compared with the control group, there were significant increases in both bone volume fraction and bone surface density. Axitinib, instead, did not show any significant improvement in osteolysis-related parameters compared with control mice (Fig. 5A and B). In line with these results, we observed a lower number of osteoclasts decorating the bone surface in cabozantinib- and lenvatinib-treated mice (Fig. 5C). These results suggest that cabozantinib and lenvatinib alone might have a beneficial effect on bone quality, an important factor to reduce skeletal-related events and preserve quality of life.

### Discontinuation of TKIs leads to exponential tumor growth and limited survival

**Figure 6:**
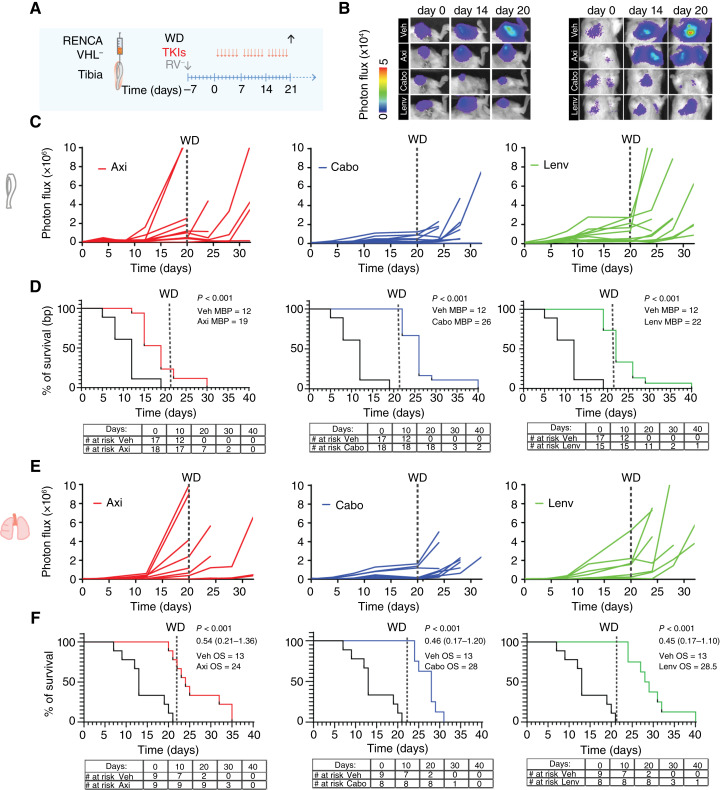
Prolonged treatment with TKIs followed by withdrawal and survival analysis. **A,** Schematic representation of experimental design. **B,** Representative images of bioluminescence signal in tibiae and lungs. **C,** Bioluminescence signal of tumors in bone quantified over time; single tumors are shown. **D,** Analysis of survival over time after TKI treatment based on bone tumors (photon flux values of 3 × 10^6^ were considered as the end point). Absolute numbers are reported in the table. *P* value (one-way ANOVA with Tukey honestly significant difference *post hoc* test) and median OS are shown. **E,** Bioluminescence signal of tumors in lungs quantified over time; **F,** Estimation of survival over time after TKI treatment based on lung tumors. Absolute numbers are reported in the table. *P* value (one-way ANOVA with Tukey honestly significant difference *post hoc* test), median OS and 95% CI of ratio after the log-rank (Mantel–Cox) test are shown. Axi, axitinib; Cabo, cabozantinib; ; Lenv, lenvatinib; MBP, median bone progression; WD, withdrawal; Veh, vehicle.

Treatment with TKIs did not lead to tumor eradication after 10 doses (Fig. 2). To investigate the durability of response in mice, we tested the impact of longer schedules of treatment (3 weeks) followed by therapy withdrawal in mice implanted with RENCA VHL^−^ cells (Fig. 6A). Even under prolonged treatment, lesions were not eradicated (Supplementary Fig. S11A and S11B). Interestingly, the axitinib-treated group showed earlier progression than the other two TKI groups in both tumor sites by the beginning of the third week of treatment. In comparison, more than 80% of tumors remained stable in both cabozantinib and lenvatinib groups until withdrawal. Eventually, all the lesions progressed following withdrawal regardless of initial treatment, and tumors progressed synchronously in both bone and lungs (Fig. 6C–F). IF analysis of blood vessels showed that tumor cell regrowth was paralleled by increasing revascularization of tumor lesions and CD8^+^ cell infiltration over time (Supplementary Fig. S12A and S12B). Despite lack of durable response, TKI-treated mice had significantly prolonged median OS (axitinib OS = 24 days, cabozantinib OS = 28 days, and lenvatinib OS = 28.5 days; *P* < 0.001) compared with control-treated mice (OS = 13 days; Fig. 6F). These results suggest that prolonged treatment with TKIs was necessary for tumor stability, whereas withdrawal led to rapid disease progression.

### Outcome of second-line treatment relies on TKIs selected in the first line

**Figure 7:**
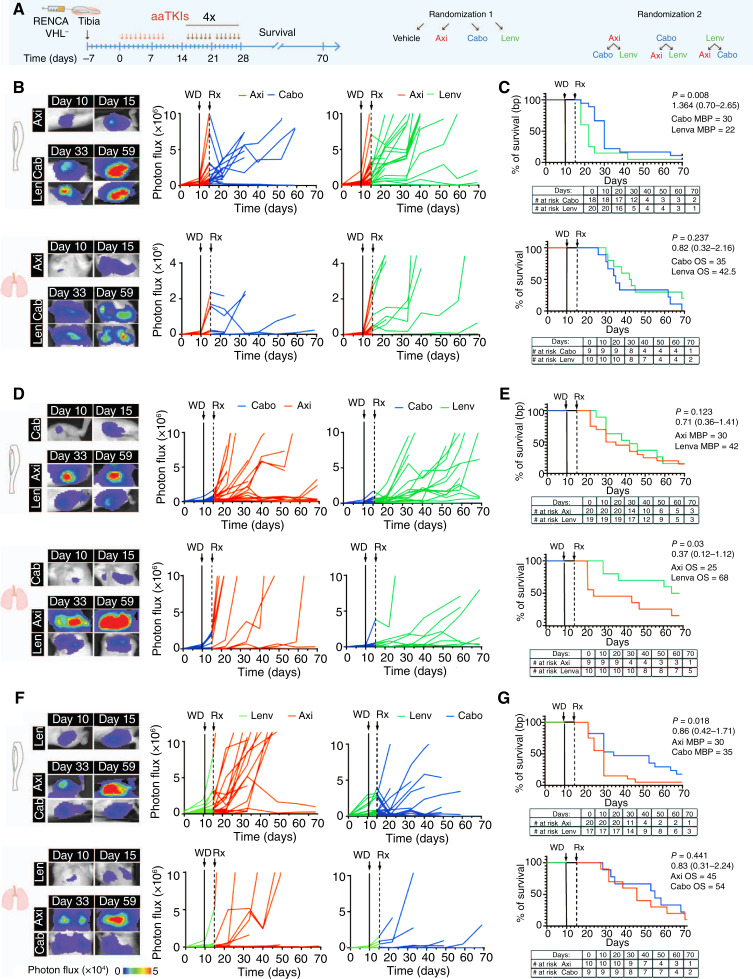
TKIs as a second-line treatment. **A,** Schematic representation of experimental design. **B**, **D**, and **F,** Representative images of bioluminescence signal in tibiae/lungs of mice and bioluminescence signal of tumors quantified over time; single tumors are shown. **C**, **E**, and **G,** Estimation of survival over time after TKI treatment based on bone and lung tumors. Absolute numbers are reported in the table. *P* value (one-way ANOVA with Tukey honestly significant difference *post hoc* test) with median OS and 95% CI of ratio after the log-rank (Mantel–Cox) test. Axi, axitinib; Cabo, cabozantinib; Lenv, lenvatinib; MBP, median bone progression; Rx, second-line treatment; WD, withdrawal.

Patients progressing on a frontline TKI are likely to be prescribed another TKI in the second or third line of therapy, but the optimal sequence of treatments is unknown. Therefore, we designed an experiment to investigate the efficacy of axitinib, cabozantinib, and lenvatinib as second-line treatments after progression on first-line treatment (Fig. 7A). The mice were treated with one of the three TKIs for 10 consecutive doses; the treatment was withdrawn, and tumor growth was monitored for 5 days. Tumors in tibiae and lungs grew four-fold during this treatment break. Axitinib-treated tumors progressed more extensively than those in the cabozantinib and lenvatinib groups in both metastatic sites, as identified by bioluminescence detection. Then, mice in each of the first-line TKI groups were randomized to two groups and received treatment with one of the two other TKIs for 6 days/week, eight cycles (up to 55 days). We found that progression in bones (median bone progression, MBP) was significantly delayed in animals receiving cabozantinib as a second-line treatment regardless of the first line. Specifically, the MBP post-axitinib was 30 days (cabozantinib) versus 22 days (lenvatinib; Fig. 7B and C), whereas the MBP post-lenvatinib was 35 days (cabozantinib) versus 30 days (axitinib; Fig. 7F and G). Additionally, sequencing cabozantinib and lenvatinib as first- and second-line treatments improved OS up to 70 days, whereas lenvatinib was better in controlling bone lesions and prolonging survival compared with axitinib following cabozantinib (axitinib—MBP = 30, OS = 25 days; lenvatinib—MBP = 42, OS = 68 days; Fig. 7D and E). However, there was no significant difference between cabozantinib and lenvatinib following axitinib in OS (cabozantinib–OS = 35 days and lenvatinib–OS = 42.5 days; Fig. 7B and C). Furthermore, axitinib was not able to control tumor progression as a second-line treatment in bone and lungs, leading to a progression of the majority of lesions after cabozantinib (OS = 35 days) or lenvatinib (OS = 45 days; Fig. 7B–G). These results suggest that specific sequences might impact outcomes, with cabozantinib followed by lenvatinib being more successful when applied sequentially than either of these drugs combined/sequenced with axitinib.

## Discussion

Axitinib, cabozantinib, and lenvatinib, the most prescribed TKIs, are currently used as first-, second-, or third-line therapy for metastatic ccRCC alone or in combination with ICIs (or everolimus for lenvatinib); however, their impact on BM has not been defined. In this work, we tested these agents in immunocompetent and immunodeficient mouse models of ccRCC in BM as well as lung metastasis, compared their efficacy as single agents, and further characterized their mechanisms of action.

In prospective clinical trials, BM are not considered measurable by RECIST v1.1 criteria unless they possess a soft tissue component, and sampling for biomarker evaluation is routinely avoided given the need for substantial processing and decalcification. As a result, ccRCC BM biology is still obscure. Therefore, utilizing preclinical models for detailed analysis of tumor response, emerging mechanisms of resistance, and potential synergistic and antagonistic effects of currently approved agents remains crucial. For this purpose, we first generated mouse models based on direct implantation of human or murine tumor cells in mouse tibia to mimic established bone lesions in patients, as our primary interest was to address the impact of TKIs on bone tumors rather than their impact on the process of metastasis development. This approach allows better control of the secondary site, consistency, and reproducibility for testing therapeutics as compared with intracardiac injection, which mimics hematogenous spread. As a limitation, our model does not recapitulate the entire metastatic cascade. On the other hand, intracardiac administration of tumor cells is nonselective and generally induces early formation of malignant lesions in soft organs (which progress rapidly and limit mouse survival) with very rare BM formation, which has mostly been found in the mandible (57). Alternatively, several transgenic models of ccRCC were recently developed (25, 27, 58–60). As an advantage, these models recapitulate major ccRCC features (e.g., similar genetic profiles of cancer cell–driving mutations, the presence of a primary tumor, metastatic presentation, etc). However, besides requiring numerous months to develop metastasis, they have relatively low penetrance, if any, and do not show bone colonization, limiting their use for drug testing and detailed characterization of therapeutic activity in bone tumors. Therefore, considering the above limitations, direct implantation of tumor cells into bone remains a key approach to test the response to therapy. Importantly, no immunocompetent models of RCC with bone tumors have been reported to date, and only one model of human ccRCC implanted in mouse tibia was established (which did not present with lung seeding; ref. 31). The RENCA cell line has limitations in accurately replicating the genetic profile of human ccRCC. However, by *vhl* gene KO, we were able to significantly improve its genetic profile, as done by others in the field (27), and enable robust and reproducible studies of the tumor niche in bone. The ability of RENCA VHL^−^ cells to colonize the lungs after injection points to intravasation into the venous system and trapping into the capillary network, a phenomenon previously described after injection into the kidney capsule or subcutaneous tissue (42, 61). This mechanism of escape from bone created the opportunity to compare two different metastatic sites simultaneously. UM-RC-3 and LVRCC67 cells (25), instead, did not establish lung tumors after intratibial injections, limiting comparison with extraosseous sites. As a future strategy to compare tumor growth and therapy response in lung and bone lesions in these models, simultaneous intratibial and i.v. injections of tumor cells can be performed.

As expected, tumor formation in our models was accompanied by progressive and extensive angiogenesis (a distinctive feature of ccRCC due to the severe impairment of the hypoxia-inducible factor signaling pathway and *vhl* mutation; ref. 62) in both bone and lung lesions. Within 1 week from administration, axitinib, cabozantinib, and lenvatinib significantly reduced tumor growth in both immunocompetent and immunodeficient mice, suggesting that their mechanisms of action are independent from the adaptive immune system, including B and T lymphocytes. Neoangiogenesis in bone and lung tumors was significantly impaired as well. Interestingly, lenvatinib showed a characteristic phenotype, with the remaining blood vessels distributed at the tumor–bone marrow interface. Axitinib-treated tumors showed revascularization by day 10 post-treatment, pointing to loss of efficacy of VEGFR inhibition, followed by consistent progression of tumor growth in bone and lungs by day 10 post-treatment. Cabozantinib and lenvatinib, instead, kept lesions stable during the treatment phase, with no animal mortality. None of the tested TKIs induced tumor eradication (even after prolonged treatment for 3 weeks), likely keeping viable tumors in a majorly avascular state in both bone and lungs, with rebound tumor growth soon after discontinuation of treatment in each TKI-treated group. Similar results were also reported for bone metastatic prostate cancer lesions after withdrawal of cabozantinib (63). Overall, this phenomenon may be due to the relatively rapid clearance of TKIs from the host (which have half-lives of 6, 28, and 99 hours for axitinib, lenvatinib, and cabozantinib, respectively, in patients; refs. 64–66) combined with the fast revascularization of these aggressive tumor cells. In line with these observations, it is interesting to note that axitinib, which has a shorter half-life, showed earlier progression (even before discontinuation). Consistently, durable remission following TKI discontinuation is not common in patients, with the majority of them progressing on TKI therapy or during treatment breaks due to toxicity or interventional procedures.

Although single-agent TKIs changed the treatment landscape of metastatic ccRCC, the use of axitinib, cabozantinib, or lenvatinib in combination with ICIs have become standard-of-care frontline regimens (21, 67, 68). Despite their broad use, mechanistic understanding of TKI impact on the bone microenvironment was lacking. Therefore, elucidating response mechanisms to individual TKIs is critical to developing optimal next-generation therapies, including approved (e.g., ICI) or experimental (e.g., belzutifan, alone or with ICI) combinations. This lack of knowledge about the mechanisms of TKIs in bone tumors includes their impact on tumor-infiltrating immune cells. Our immunocompetent model of ccRCC in bone allowed for analysis of the immune infiltrate and changes resulting from systemic therapy. Treatment-naïve tumors, both in bone and lungs, showed an abundance of CD8^+^ T cells, in line with published evidence of high T-cell infiltration in ccRCC (69). We found a prominent impact of TKIs on immune infiltrating cells, which appeared to be linked directly to tumor blood vessel reduction. The total number of CD8^+^ lymphocytes associated with tumors in bone and lung lesions was indeed diminished. This reduction was directly proportional to tumor dimensions, with no difference in the local density of these cells in bone tumors. However, TKIs significantly impacted the distribution of the CD8^+^ lymphocytes, which mostly localized at the tumor interface with bone marrow. These results were confirmed in LVRCC67 bone lesions, suggesting consistent mechanisms of action for TKIs across different models. This phenomenon was not only limited to CD8^+^ cells but was confirmed in other lymphoid and myeloid cell types, with cabozantinib and lenvatinib limiting infiltration of the tumor. Limitations imposed by TKIs in immune cell infiltration might significantly impact combinational treatments with ICIs. Interestingly, a previous study in mice injected with RENCA cells intravenously and treated with axitinib and an anti-PD1 antibody found that mouse survival was influenced by the sequence of treatments. Axitinib treatment followed by anti-PD1 led to shorter survival than concurrent treatment or administration of anti-PD1 first followed by axitinib (51). Although this study did not provide mechanistic results, this evidence is in line with our results about the impact of TKIs on infiltrating immune cells in bone lesions, which could limit second- and later-line ICI efficacy (67). Consequently, the sequence of agents in combination regimens should be rationally designed for the treatment of BM.

The majority of patients who receive a TKI and progress on therapy are likely to receive another TKI in second- or third-line therapy. Therefore, rational sequencing of TKIs is of importance. A phase III study of axitinib versus sorafenib in the second-line treatment setting found improvement in PFS for patients treated with axitinib (70). It was followed with approval of cabozantinib and lenvatinib with everolimus in the post-TKI setting (13, 14). However, there is no current available head-to-head data comparing these regimens. Our hypothesis-generating experiments on the sequencing of TKI treatment suggest that axitinib is not equally potent as a second-line option compared with cabozantinib or lenvatinib and is unlikely to be an effective salvage strategy. Furthermore, they highlight that cabozantinib and lenvatinib can alter the tumor microenvironment in a way that may lead to a differential response (and resistance) to subsequent lines of therapy. Interestingly, sequencing cabozantinib and lenvatinib as a first- and second-line treatment showed to be the most durable regarding PFS and OS in mice with bone and lung tumors.

BM in ccRCC exhibits extensive osteolysis, which is the cause of significant pain and paraneoplastic episodes such as pathologic fractures and skeletal-related events (37). Historically, patients with ccRCC BM are treated with bone modifying agents, like denosumab or zoledronic acid; however, the role of these agents has been brought into question (37, 71). Since the era of targeted therapies, including anti-VEGFR TKIs tested in our study, numerous benefits about slowed progression of BM were noted. However, the combination of these drugs with bisphosphonates increased the risk of osteonecrosis of the jaw up to 17% (72). Our models presented with an osteolytic phenotype, a clinically relevant aspect of this disease. Cabozantinib and lenvatinib significantly limited bone resorption in VHL^−^ RENCA-bearing tumors, as previously identified in cabozantinib-treated RCC 786-O bone tumors (31), suggesting a mechanism for TKIs alone to reduce skeletal-related events, as found in the subgroup analysis of the METEOR study (73). Likely, these effects are a consequence of tumor reduction, which might impact recruitment and activation of osteoclasts. Further studies are needed to rule out whether TKIs might have a direct effect on osteoclast biology.

In conclusion, using our newly developed immunocompetent and immunodeficient mouse models of established ccRCC bone lesions, we were able to dissect the therapeutic response to TKIs, and this allows us to explore future preclinical development of combination therapies to inform further development of highly effective therapeutic strategies, especially against ccRCC in bone.

## Supplementary Material

Figure S1 Blood vessel marker expression in bone marrow and tumors. A, B) Detection of bone marrow (A) and tumor (B) blood vessels by confocal acquisition; blue, DAPI; yellow, endomucin; red, laminin; green, tumor GFP; white boxes, magnifications reported on right panels; arrowhead, lack of physical overlap between markers; C) Immunofluorescence detection of blood vessels in VHL- RENCA bone tumors by confocal microscopy; blue, DAPI; yellow, endomucin; red, laminin; green, tumor GFP; D) Quantification of blood vessel area inside and outside tumor area based on laminin (red) and endomucin (yellow) signal, mean + SEM, n=3-4/group; blue, DAPI; green, GFP; arrowhead, blood vessels with only one marker expressed. Bar, 10 µm. Endo-endomucin; Lam-laminin; IN T-inside tumor; OUT T-outside tumor.

Figure S2 Osteoclast recruitment and bone disruption. A) RENCA VHL- cells (green, GFP); osteoclasts (red, TRAP); nuclei (blue, DAPI); white arrowhead, TRAP+ cells. Bar, 50 µm; B) UM-RC-3 (green, GFP); osteoclasts (red, TRAP); nuclei (blue, DAPI); white arrowhead, TRAP+ cells. Bar, 50 µm. Images captured by confocal microscope. TRAP-tartrate-resistant acid phosphatase. White rectangle, (i); magnification.

Figure S3 Efficacy of TKIs in VHL- RENCA tumors treated at day 14 post-tumor injection. A) Visual representation of the experimental approach. B) Representative images of bioluminescence signal in bones followed by quantification of photon flux, mean + SEM, n=8-16/group; P values by one-way ANOVA with Tukey’s HSD post hoc test; p < 0.0001 = ****.

Figure S4 Effects of TKIs on blood vessels in RENCA VHL- bone and lung tumors. A) Visual representation of the experimental approach. B,C) Bone tumors: representative pictures captured at the confocal microscope and quantification of blood vessel area in tumor (green, GFP), nuclei (blue, DAPI); blood vessels (yellow, endomucin; red, laminin); dotted line, tumor edge; single channel endomucin is shown. A quantification of % blood vessels on tumor area is shown, mean + SEM, n=3-4/group; Bar, 100 µm. D,E) Lung tumors: representative pictures captured at the confocal microscope and quantification (C) of blood vessel area in tumor (green, GFP), nuclei (blue, DAPI); blood vessels (red, CD31); dotted line, tumor edge; single channel CD31 is shown. A quantification of % blood vessels on tumor area is shown, mean + SEM, n=3-4/group; Bar, 100 µm P values by one-way ANOVA with Tukey’s HSD post hoc test; p < 0.05 = *, p < 0.01 = **. BV- blood vessels. Endo-endomucin; Lam-laminin; BV-blood vessels; V-vehicle, A-axitinib, C-cabozantinib, L-lenvatinib;

Figure S5 Cross-sectional slices of tibiae with VHL- RENCA after TKIs treatment. Representative images of bone tumors post- treatment, obtained by confocal microscope: green, GFP; yellow, endomucin; red, laminin; blue, DAPI. Bar, 100 µm. Endo-endomucin; Lam-laminin.

Figure S6 Blood vessel distance analysis in bone tumors. (1) Representative images of bone (VHL- RENCA) tumors captured by confocal microscope; tumor cells (green, GFP); blood vessels (yellow, endomucin; red, laminin), nuclei (blue, DAPI); (2) single endomucin channel and examples of measures (yellow bar lines) of blood vessel distance calculated in Fig. 3 D, F; dotted line, the area of tumor. Endo-endomucin; Lam-laminin.

Figure S7 Effects of TKIs on bone tumors in UM-RC-3 model. A) Visual representation of the experimental approach. B, C) Neo-angiogenesis in tibiae (B) and quantification (C) of area occupied by blood vessels in tumors; representative images obtained by confocal microscope, blue, DAPI; green, GFP; yellow, endomucin; red, laminin; dotted line, tumor edge; mean + SEM, n=4/group. P values by one-way ANOVA with Tukey’s HSD post hoc test; p < 0.05 = *, p < 0.01 = **. Bar, 100 µm. Endo/Endom-endomucin; Lam-laminin; BV-blood vessels; V-vehicle, A-axitinib, C-cabozantinib, L-lenvatinib.

Figure S8 Analysis of CD8+ cell infiltration at baseline in bone tumors and description of COMET analysis. A) Representative images captured by confocal microscope of CD8+ cell infiltration in bone tumors and quantification of CD8+ cell number; tumor (green, GFP), CD8+ cells (yellow), blood vessels (laminin, red), nuclei (blue, DAPI); dashed line, tumor edge; mean + SEM, n=3/group; dotted lines. B) Spatial definition of tumor edge; dashed line, tumor edge; dotted lines were draw at + 25 µm of distance from the tumor edge, that represents the tumor interface. C) A quantification of the % of CD8+ cells is shown, mean + SEM, n=3/group. D) Visual representation of experimental design (referred to Figure 4.). E, F) Representative images captured by confocal microscope of the distance (dotted line) between CD8+ cells (yellow) and the closest blood vessel (red, laminin) with quantification, mean + SEM, n=25-75/group. (F). G) Representation of step-by-step process of analysis of the immune infiltrate including tissue segmentation in VisioPharm. P values by one-way ANOVA with Tukey’s HSD post hoc test; p < 0.05 = *. Bar, 25 µm.Veh- vehicle; Axi- axitinib; Cabo- cabozantinib; Lenv- lenvatinib. Lam-laminin; Inner reg-inner region.

Figure S9 CD8+ cell infiltration of lung tumors at baseline and following TKI administration. A) Representative images of CD8+ cells in lung tumors captured by confocal microscope at baseline (no treatment), at day 7, 14 and 21 post-tumor cell injection; tumor (green, GFP), CD8+ cells (yellow), blood vessels (CD31, red), nuclei (blue, DAPI); dotted line, tumor edge. B) A quantification of CD8+ cell number and their distribution over time is shown, mean + SEM, n=3/group. C) Representative images of CD8+ cells in lung tumors captured by confocal microscope post-TKI treatment; tumor (green, GFP); of CD8+ cells (yellow), blood vessels (red, CD31), nuclei (blue, DAPI); dotted line, tumor edge. D) A quantification of CD8+ cell number, number of CD8+ cells over tumor area and CD8+ cell distribution is shown; mean + SEM, n=6-8/group. P values by one-way ANOVA with Tukey’s HSD post hoc test; p < 0.05 = *, p < 0.01 = **. Bar, 100 µm. V-vehicle, A-axitinib, C-cabozantinib, L-lenvatinib; Inner reg-inner region.

Figure S10 TKIs’ effect on blood vessels and immune infiltrate in LVRCC67 model. A) Representative images of LVRCC67 bone tumors captured by confocal microscope; tumor cells (green, PAX8), of CD8+ cells (yellow), blood vessels (CD31, red); nuclei (blue, DAPI); dotted line, tumor edge; B) Quantification of blood vessel area, number of CD8+ cells over tumor area and CD8+ cell distribution, mean + SEM, n=3/group. P values by one-way ANOVA with Tukey’s HSD post hoc test; p < 0.01 = **. Bar, 100 µm. BV-blood vessels; V-vehicle, A-axitinib, C-cabozantinib, L-lenvatinib; Inner reg-inner region.

Figure S11 In vivo efficacy of TKIs in VHL- RENCA model over 20 days. A, B) Response to TKIs in bone (A) and lung (B) tumors detected by bioluminescence, mean + SEM, n = 8 to 16/group.

Figure S12 Effects of TKI withdrawal on vascularization and immune cell infiltration in bone tumors. A) Representative images of bone tumors 12 days following withdrawal of TKIs; tumor (green, GFP), blood vessels (red, laminin) nuclei (blue, DAPI) and CD8+ cells (yellow) images captured by confocal microscope and quantifications are shown (B); mean + SEM, n=3/group. Bar, 100 µm. BV – blood vessels. Lam-laminin; IN-inner region.
