# Supplementary figures and images for: Antiangiogenic Tyrosine Kinase Inhibitors have Differential Efficacy in Clear Cell Renal Cell Carcinoma in Bone
Source: Cancer Res Commun. 2024 Oct 8;4(10):2621–37. doi: 10.1158/2767-9764.CRC-24-0304 (PMC11459607; doi:10.1158/2767-9764.CRC-24-0304)

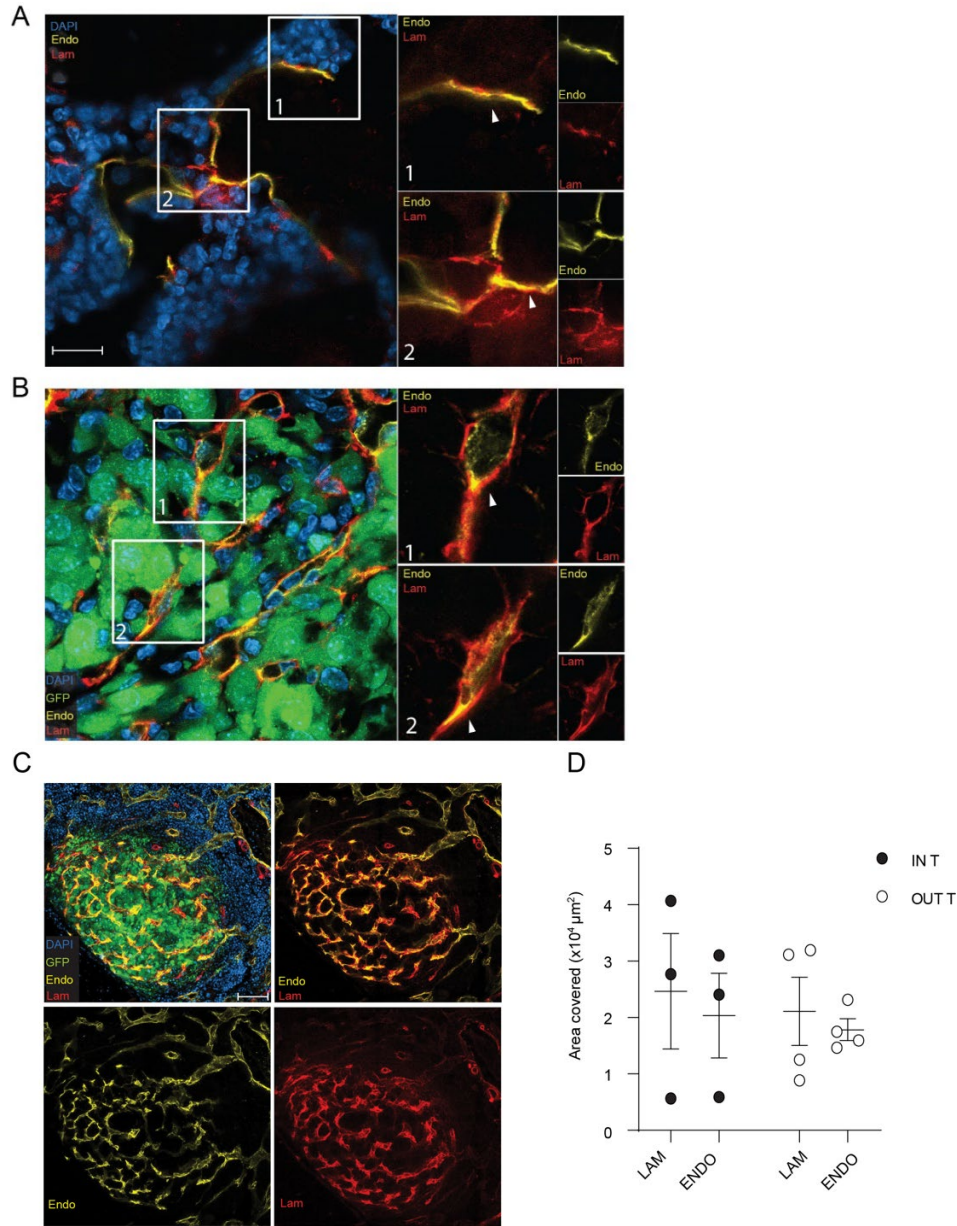

Supplement: Figure S1 — Blood vessel marker expression in bone marrow and tumors. A, B) Detection of bone marrow (A) and tumor (B) blood vessels by confocal acquisition; blue, DAPI; yellow, endomucin; red, laminin; green, tumor GFP; white boxes, magnifications reported on right panels; arrowhead, lack of physical overlap between markers; C) Immunofluorescence detection of blood vessels in VHL- RENCA bone tumors by confocal microscopy; blue, DAPI; yellow, endomucin; red, laminin; green, tumor GFP; D) Quantification of blood vessel area inside and outside tumor area based on laminin (red) and endomucin (yellow) signal, mean + SEM, n=3-4/group; blue, DAPI; green, GFP; arrowhead, blood vessels with only one marker expressed. Bar, 10 µm. Endo-endomucin; Lam-laminin; IN T-inside tumor; OUT T-outside tumor. [file crc-24-0304_figure_s1_suppsf1.pdf]

A

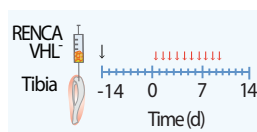

B

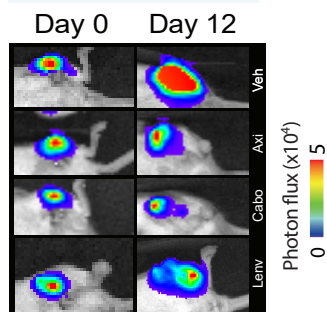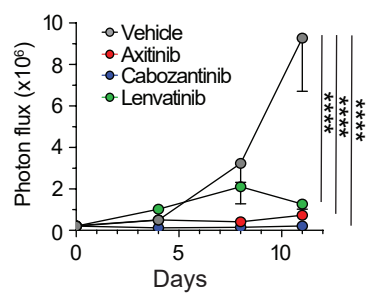

Supplement: Figure S3 — Efficacy of TKIs in VHL- RENCA tumors treated at day 14 post-tumor injection. A) Visual representation of the experimental approach. B) Representative images of bioluminescence signal in bones followed by quantification of photon flux, mean + SEM, n=8-16/group; P values by one-way ANOVA with Tukey’s HSD post hoc test; p < 0.0001 = ****. [file crc-24-0304_figure_s3_suppsf3.pdf]

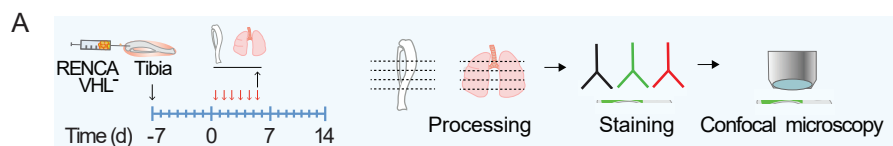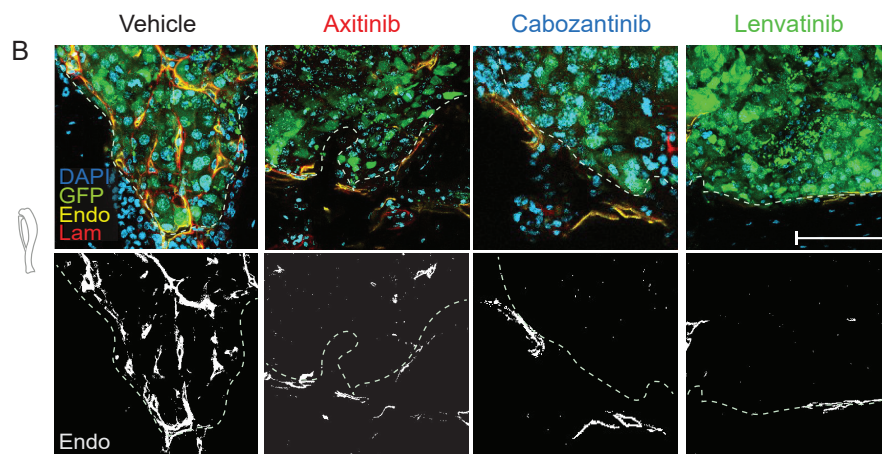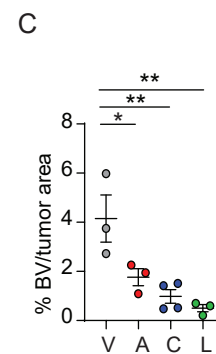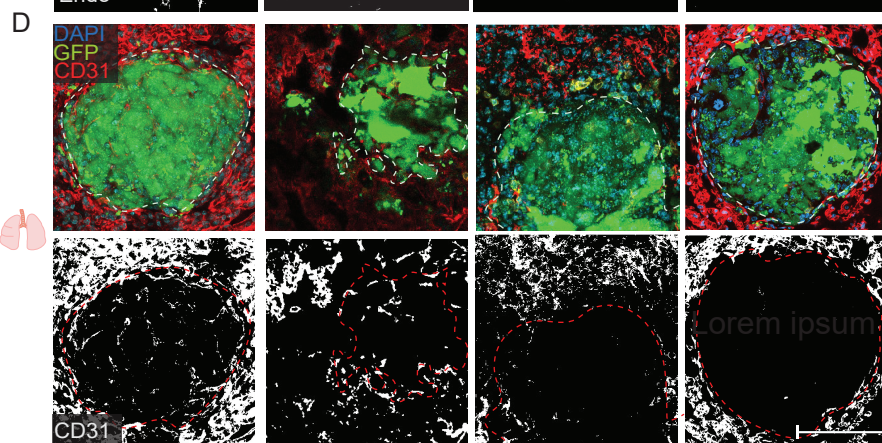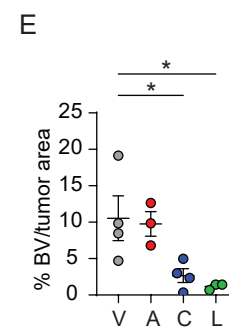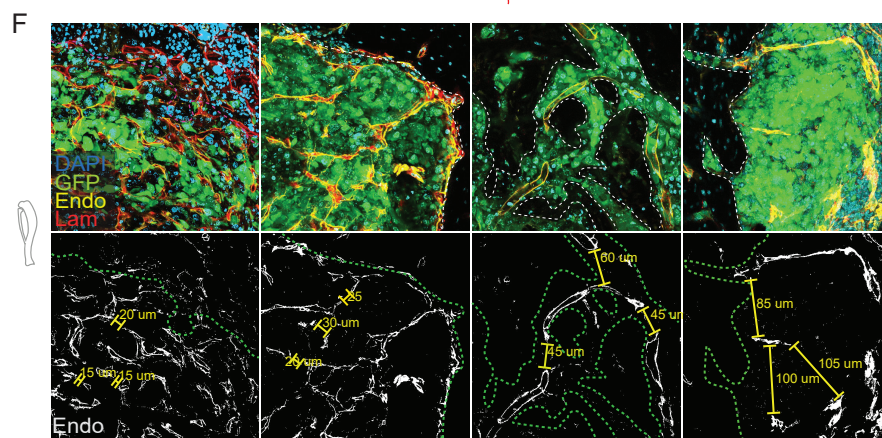

Supplement: Figure S4 — Effects of TKIs on blood vessels in RENCA VHL- bone and lung tumors. A) Visual representation of the experimental approach. B,C) Bone tumors: representative pictures captured at the confocal microscope and quantification of blood vessel area in tumor (green, GFP), nuclei (blue, DAPI); blood vessels (yellow, endomucin; red, laminin); dotted line, tumor edge; single channel endomucin is shown. A quantification of % blood vessels on tumor area is shown, mean + SEM, n=3-4/group; Bar, 100 µm. D,E) Lung tumors: representative pictures captured at the confocal microscope and quantification (C) of blood vessel area in tumor (green, GFP), nuclei (blue, DAPI); blood vessels (red, CD31); dotted line, tumor edge; single channel CD31 is shown. A quantification of % blood vessels on tumor area is shown, mean + SEM, n=3-4/group; Bar, 100 µm P values by one-way ANOVA with Tukey’s HSD post hoc test; p < 0.05 = *, p < 0.01 = **. BV- blood vessels. Endo-endomucin; Lam-laminin; BV-blood vessels; V-vehicle, A-axitinib, C-cabozantinib, L-lenvatinib; [file crc-24-0304_figure_s4_suppsf4.pdf]

Vehicle

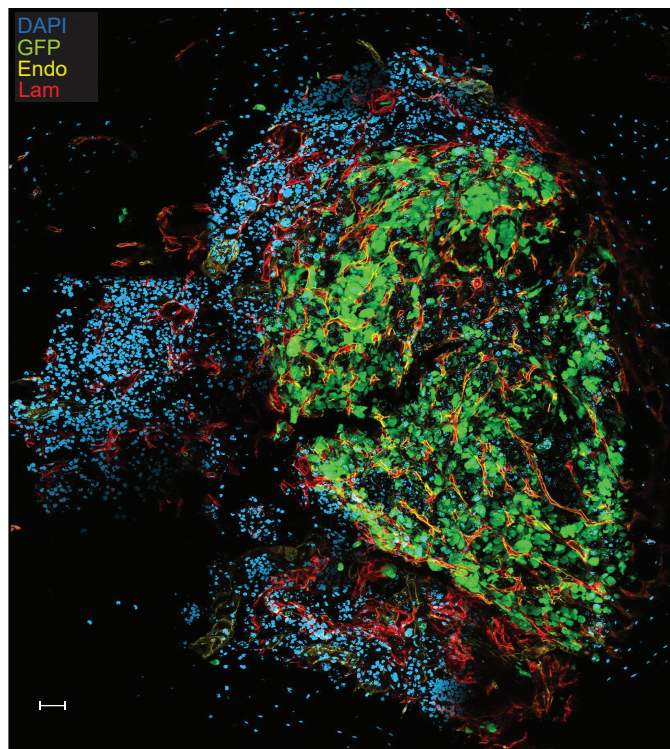

Axitinib

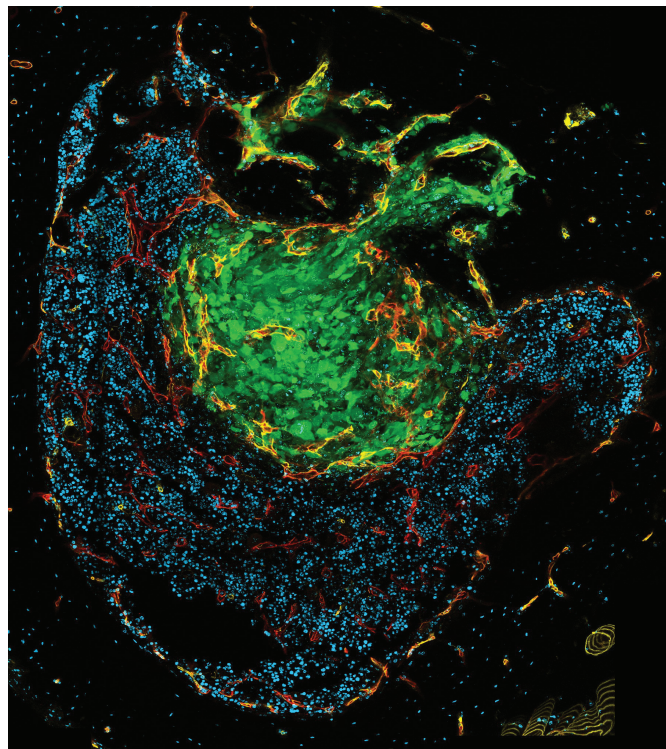

Cabozantinib

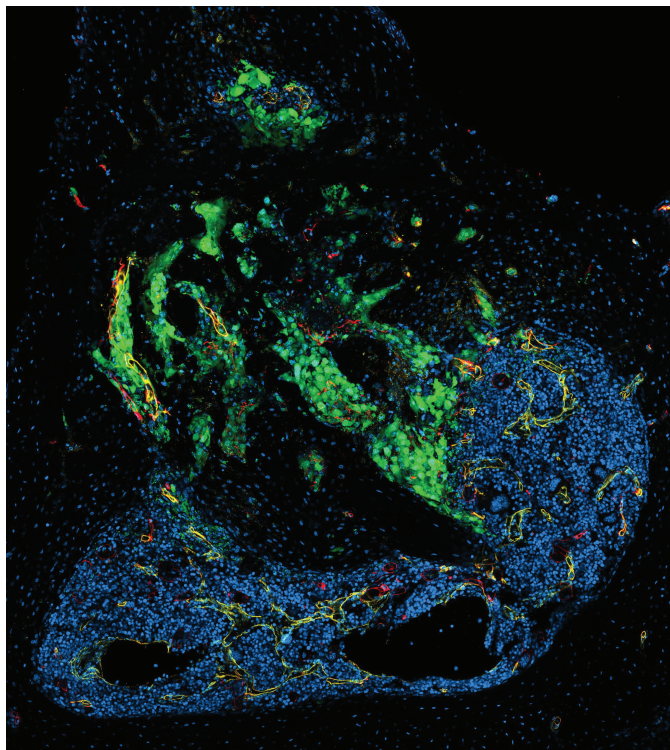

Lenvatinib

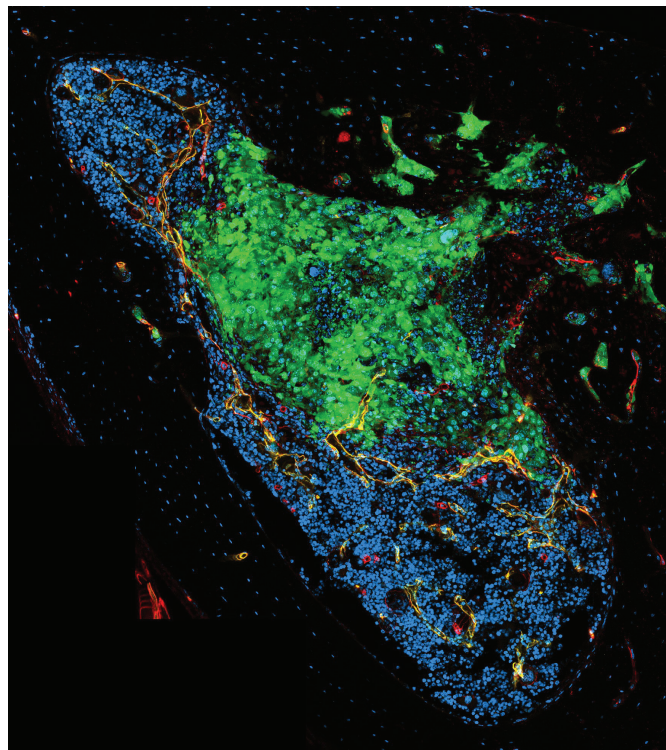

Supplement: Figure S5 — Cross-sectional slices of tibiae with VHL- RENCA after TKIs treatment. Representative images of bone tumors post- treatment, obtained by confocal microscope: green, GFP; yellow, endomucin; red, laminin; blue, DAPI. Bar, 100 µm. Endo-endomucin; Lam-laminin. [file crc-24-0304_figure_s5_suppsf5.pdf]

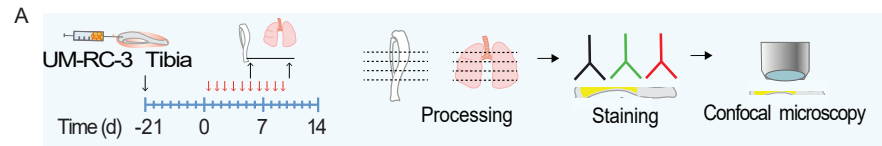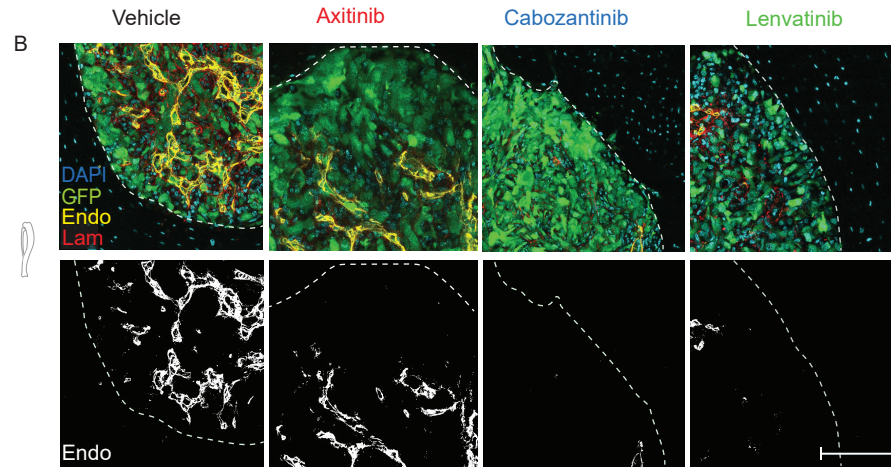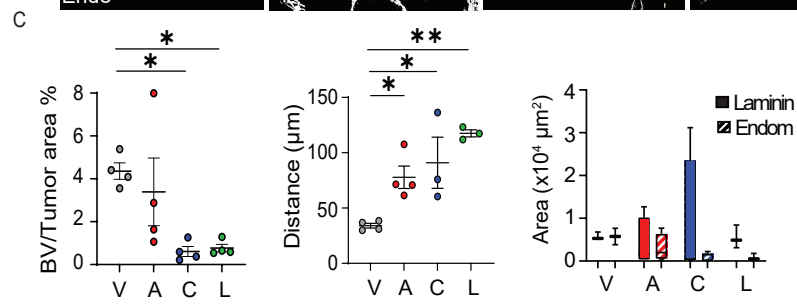

Supplement: Figure S7 — Effects of TKIs on bone tumors in UM-RC-3 model. A) Visual representation of the experimental approach. B, C) Neo-angiogenesis in tibiae (B) and quantification (C) of area occupied by blood vessels in tumors; representative images obtained by confocal microscope, blue, DAPI; green, GFP; yellow, endomucin; red, laminin; dotted line, tumor edge; mean + SEM, n=4/group. P values by one-way ANOVA with Tukey’s HSD post hoc test; p < 0.05 = *, p < 0.01 = **. Bar, 100 µm. Endo/Endom-endomucin; Lam-laminin; BV-blood vessels; V-vehicle, A-axitinib, C-cabozantinib, L-lenvatinib. [file crc-24-0304_figure_s7_suppsf7.pdf]

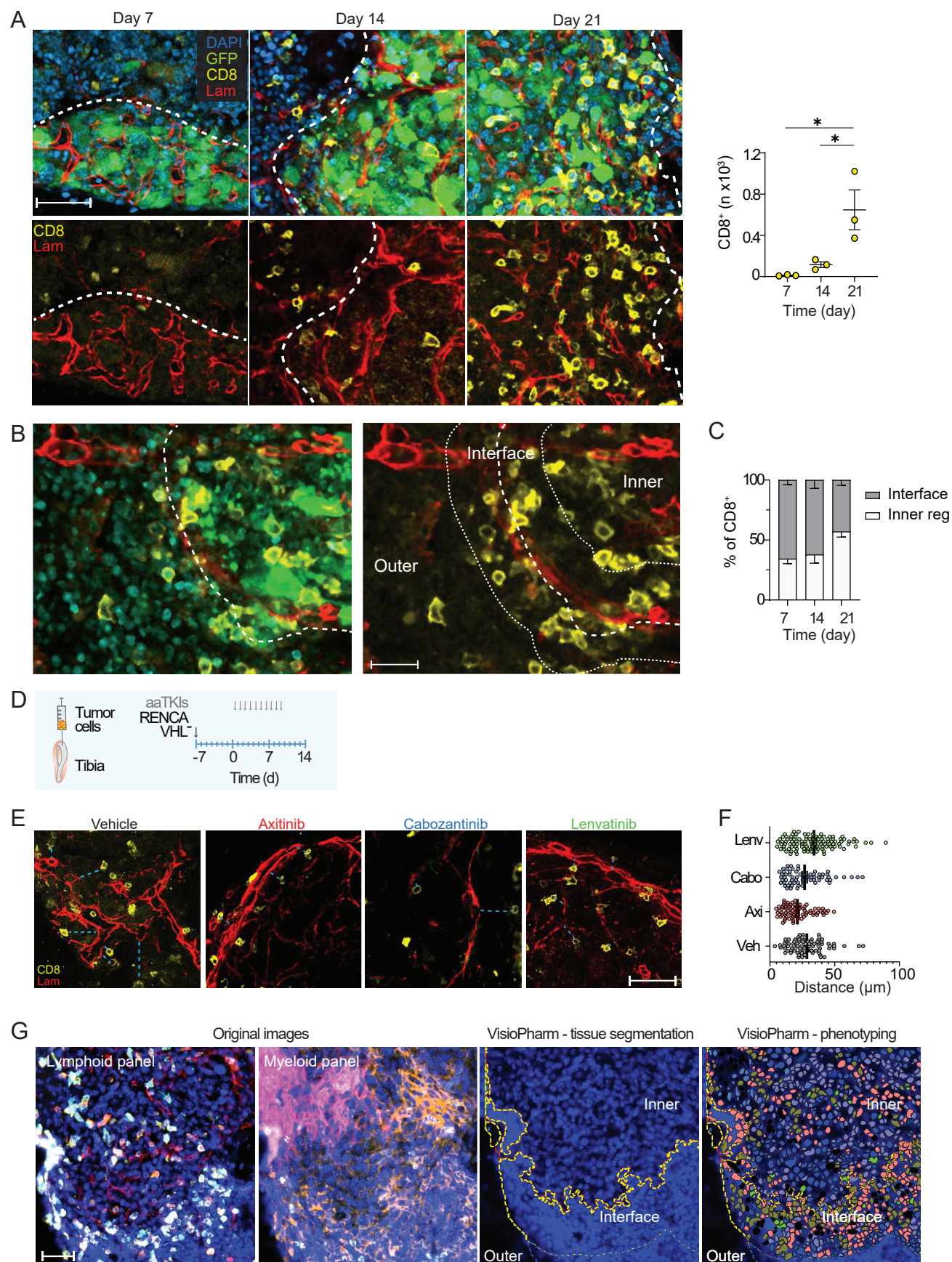

Supplement: Figure S8 — Analysis of CD8+ cell infiltration at baseline in bone tumors and description of COMET analysis. A) Representative images captured by confocal microscope of CD8+ cell infiltration in bone tumors and quantification of CD8+ cell number; tumor (green, GFP), CD8+ cells (yellow), blood vessels (laminin, red), nuclei (blue, DAPI); dashed line, tumor edge; mean + SEM, n=3/group; dotted lines. B) Spatial definition of tumor edge; dashed line, tumor edge; dotted lines were draw at + 25 µm of distance from the tumor edge, that represents the tumor interface. C) A quantification of the % of CD8+ cells is shown, mean + SEM, n=3/group. D) Visual representation of experimental design (referred to Figure 4.). E, F) Representative images captured by confocal microscope of the distance (dotted line) between CD8+ cells (yellow) and the closest blood vessel (red, laminin) with quantification, mean + SEM, n=25-75/group. (F). G) Representation of step-by-step process of analysis of the immune infiltrate including tissue segmentation in VisioPharm. P values by one-way ANOVA with Tukey’s HSD post hoc test; p < 0.05 = *. Bar, 25 µm.Veh- vehicle; Axi- axitinib; Cabo- cabozantinib; Lenv- lenvatinib. Lam-laminin; Inner reg-inner region. [file crc-24-0304_figure_s8_suppsf8.pdf]

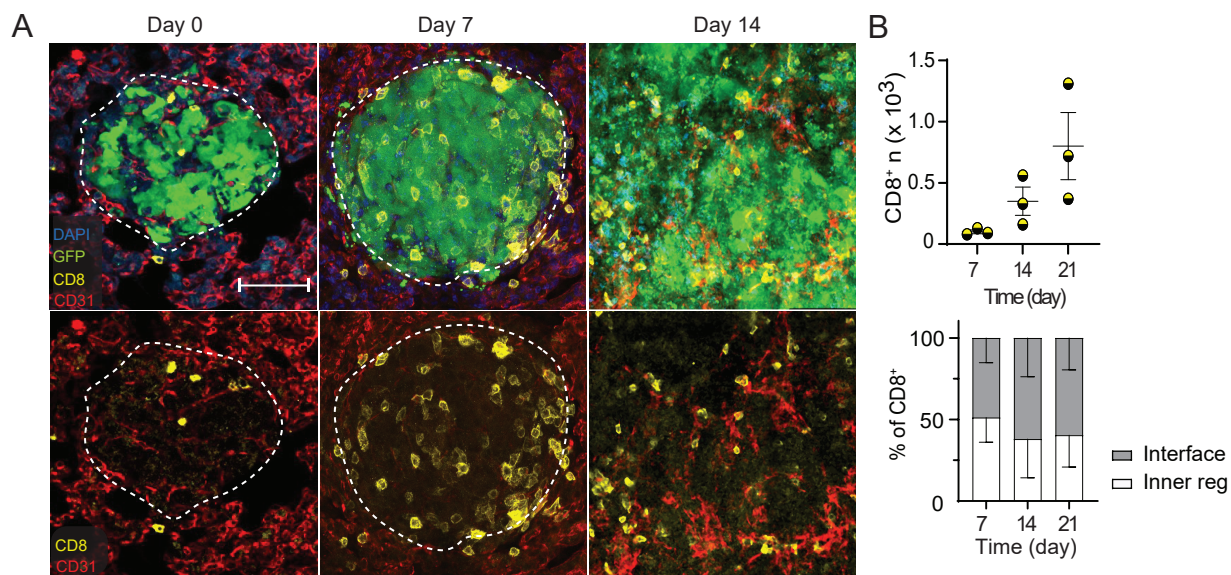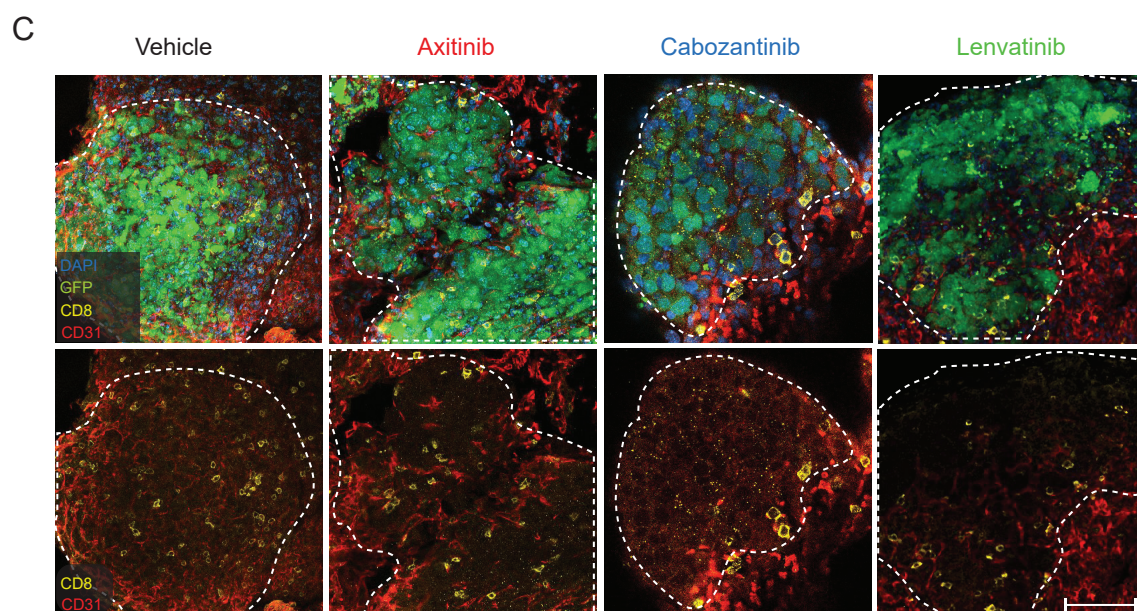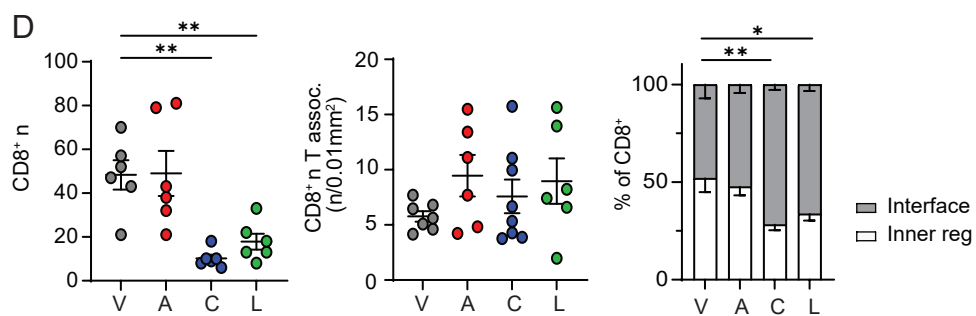

Supplement: Figure S9 — CD8+ cell infiltration of lung tumors at baseline and following TKI administration. A) Representative images of CD8+ cells in lung tumors captured by confocal microscope at baseline (no treatment), at day 7, 14 and 21 post-tumor cell injection; tumor (green, GFP), CD8+ cells (yellow), blood vessels (CD31, red), nuclei (blue, DAPI); dotted line, tumor edge. B) A quantification of CD8+ cell number and their distribution over time is shown, mean + SEM, n=3/group. C) Representative images of CD8+ cells in lung tumors captured by confocal microscope post-TKI treatment; tumor (green, GFP); of CD8+ cells (yellow), blood vessels (red, CD31), nuclei (blue, DAPI); dotted line, tumor edge. D) A quantification of CD8+ cell number, number of CD8+ cells over tumor area and CD8+ cell distribution is shown; mean + SEM, n=6-8/group. P values by one-way ANOVA with Tukey’s HSD post hoc test; p < 0.05 = *, p < 0.01 = **. Bar, 100 µm. V-vehicle, A-axitinib, C-cabozantinib, L-lenvatinib; Inner reg-inner region. [file crc-24-0304_figure_s9_suppsf9.pdf]

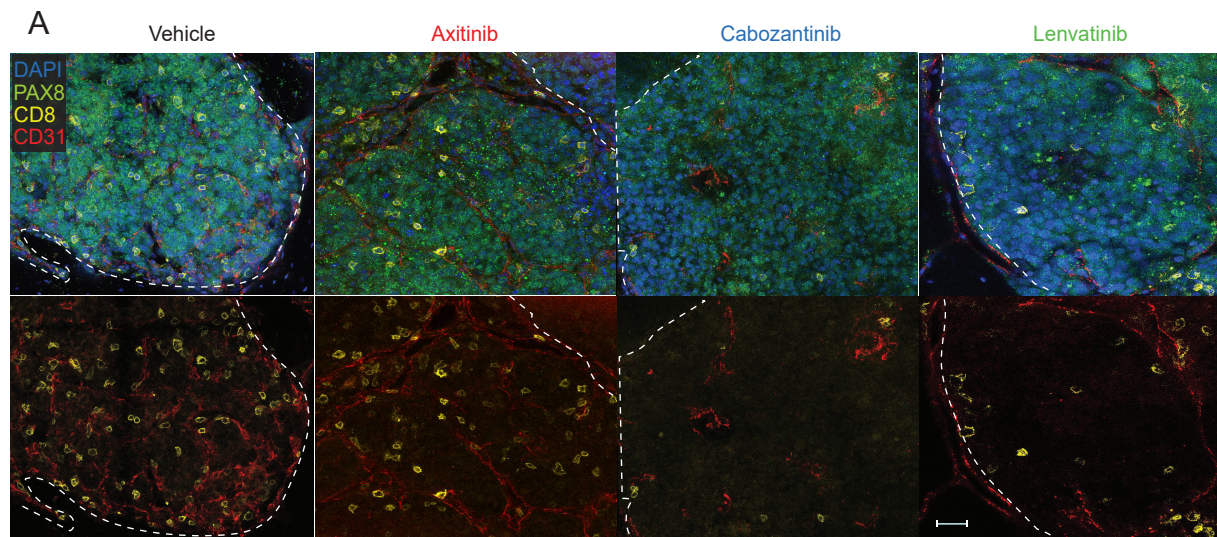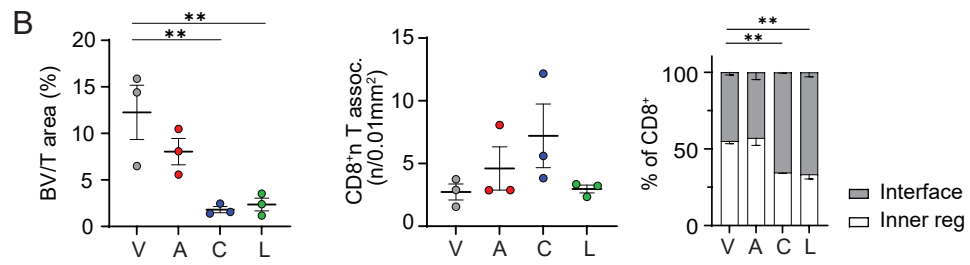

Supplement: Figure S10 — TKIs’ effect on blood vessels and immune infiltrate in LVRCC67 model. A) Representative images of LVRCC67 bone tumors captured by confocal microscope; tumor cells (green, PAX8), of CD8+ cells (yellow), blood vessels (CD31, red); nuclei (blue, DAPI); dotted line, tumor edge; B) Quantification of blood vessel area, number of CD8+ cells over tumor area and CD8+ cell distribution, mean + SEM, n=3/group. P values by one-way ANOVA with Tukey’s HSD post hoc test; p < 0.01 = **. Bar, 100 µm. BV-blood vessels; V-vehicle, A-axitinib, C-cabozantinib, L-lenvatinib; Inner reg-inner region. [file crc-24-0304_figure_s10_suppsf10.pdf]

Day 32 (12 days following withdrawal)

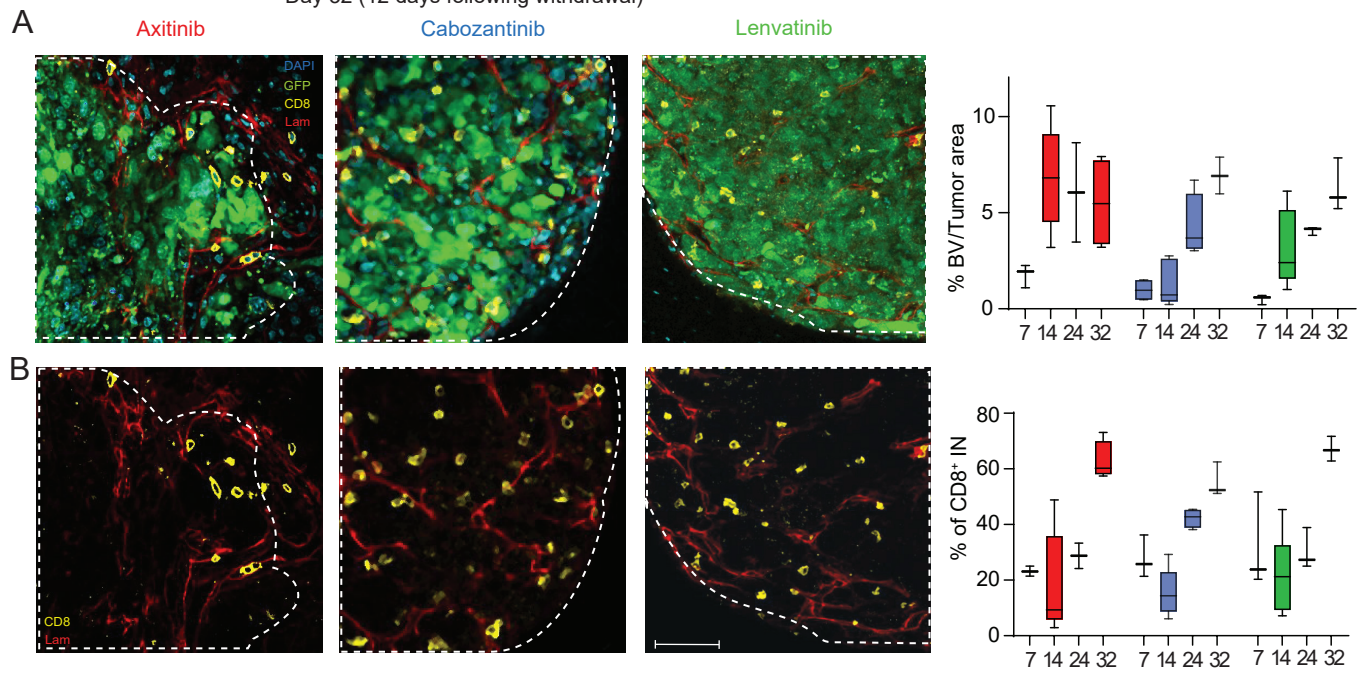

Supplement: Figure S12 — Effects of TKI withdrawal on vascularization and immune cell infiltration in bone tumors. A) Representative images of bone tumors 12 days following withdrawal of TKIs; tumor (green, GFP), blood vessels (red, laminin) nuclei (blue, DAPI) and CD8+ cells (yellow) images captured by confocal microscope and quantifications are shown (B); mean + SEM, n=3/group. Bar, 100 µm. BV – blood vessels. Lam-laminin; IN-inner region. [file crc-24-0304_figure_s12_suppsf12.pdf]
