# Supplementary material for: Antiangiogenic Tyrosine Kinase Inhibitors have Differential Efficacy in Clear Cell Renal Cell Carcinoma in Bone
Source: Cancer Res Commun. 2024 Oct 8;4(10):2621–37. doi: 10.1158/2767-9764.CRC-24-0304 (PMC11459607; doi:10.1158/2767-9764.CRC-24-0304)
Supplement: Figure S2 — Osteoclast recruitment and bone disruption. A) RENCA VHL- cells (green, GFP); osteoclasts (red, TRAP); nuclei (blue, DAPI); white arrowhead, TRAP+ cells. Bar, 50 µm; B) UM-RC-3 (green, GFP); osteoclasts (red, TRAP); nuclei (blue, DAPI); white arrowhead, TRAP+ cells. Bar, 50 µm. Images captured by confocal microscope. TRAP-tartrate-resistant acid phosphatase. White rectangle, (i); magnification. [file crc-24-0304_figure_s2_suppsf2.pdf]

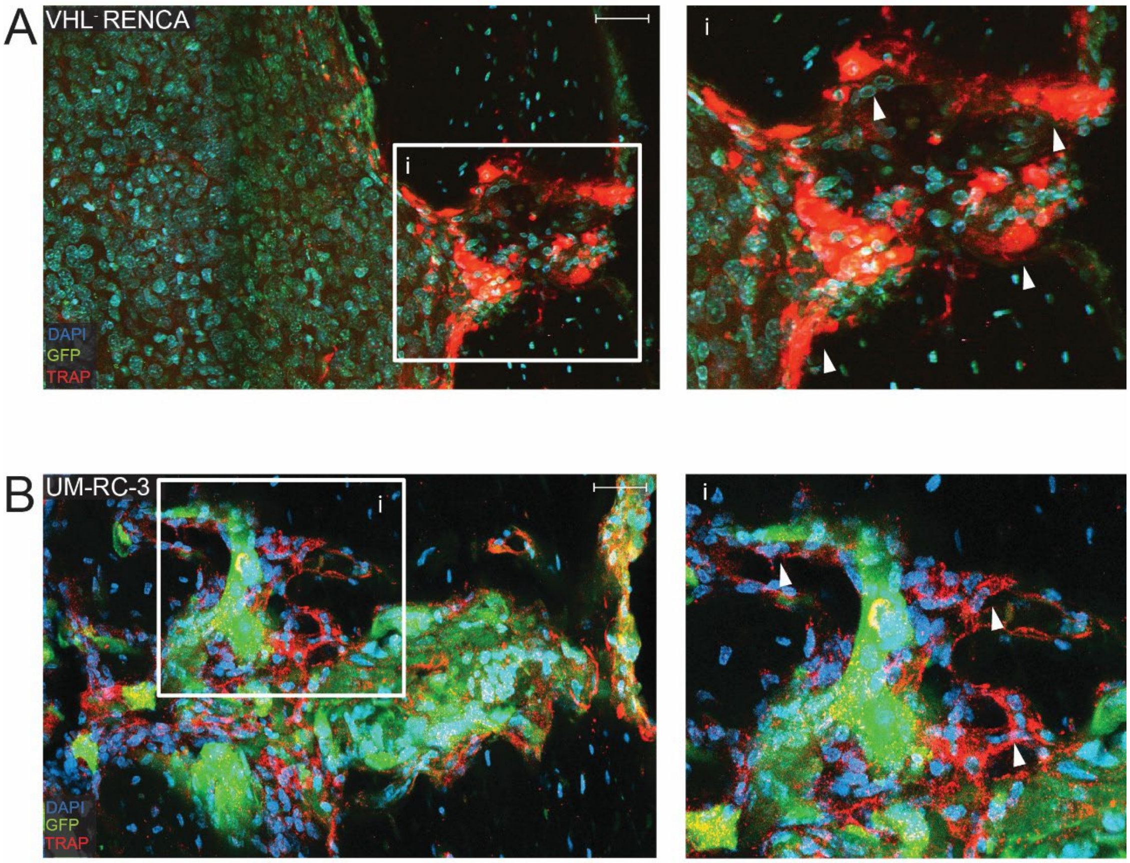

Figure S2. Osteoclast recruitment and bone disruption. A) RENCA VHL$^-$ cells (green, GFP); osteoclasts (red, TRAP); nuclei (blue, DAPI); white arrowhead, TRAP$^+$ cells. Bar, 50 μm; **B)** UM-RC-3 (green, GFP)**;** osteoclasts (red, TRAP); nuclei (blue, DAPI); white arrowhead, TRAP$^+$ cells. Bar, 50 μm. Images captured by confocal microscope. TRAP-tartrate-resistant acid phosphatase. White rectangle, (i); magnification.
