# Supplementary material for: Antiangiogenic Tyrosine Kinase Inhibitors have Differential Efficacy in Clear Cell Renal Cell Carcinoma in Bone
Source: Cancer Res Commun. 2024 Oct 8;4(10):2621–37. doi: 10.1158/2767-9764.CRC-24-0304 (PMC11459607; doi:10.1158/2767-9764.CRC-24-0304)
Supplement: Figure S6 — Blood vessel distance analysis in bone tumors. (1) Representative images of bone (VHL- RENCA) tumors captured by confocal microscope; tumor cells (green, GFP); blood vessels (yellow, endomucin; red, laminin), nuclei (blue, DAPI); (2) single endomucin channel and examples of measures (yellow bar lines) of blood vessel distance calculated in Fig. 3 D, F; dotted line, the area of tumor. Endo-endomucin; Lam-laminin. [file crc-24-0304_figure_s6_suppsf6.pdf]

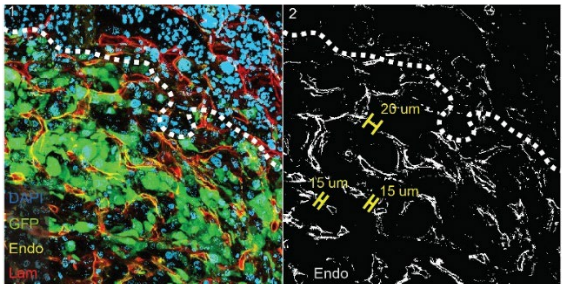

Figure S6. Blood vessel distance analysis in bone tumors. (1) Representative images of bone (VHL^{-} RENCA) tumors captured by confocal microscope; tumor cells (green, GFP); blood vessels (yellow, endomucin; red, laminin), nuclei (blue, DAPI); (2) single endomucin channel and examples of measures (yellow bar lines) of blood vessel distance calculated in **Fig. 3 D, F**; dotted line, the area of tumor. Endo-endomucin; Lam-laminin.
