# Supplementary material for: Antiangiogenic Tyrosine Kinase Inhibitors have Differential Efficacy in Clear Cell Renal Cell Carcinoma in Bone
Source: Cancer Res Commun. 2024 Oct 8;4(10):2621–37. doi: 10.1158/2767-9764.CRC-24-0304 (PMC11459607; doi:10.1158/2767-9764.CRC-24-0304)
Supplement: Figure S11 — In vivo efficacy of TKIs in VHL- RENCA model over 20 days. A, B) Response to TKIs in bone (A) and lung (B) tumors detected by bioluminescence, mean + SEM, n = 8 to 16/group. [file crc-24-0304_figure_s11_suppsf11.pdf]

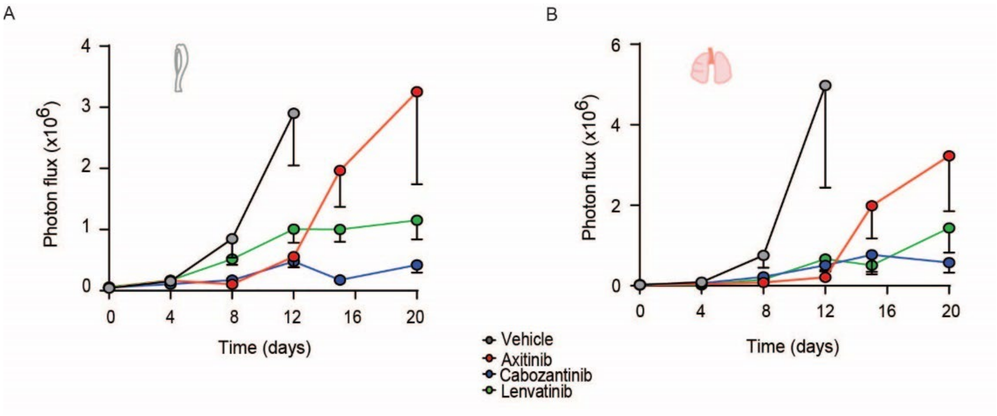

Figure S11. ***In vivo*** **efficacy of TKIs in VHL^{-} RENCA model over 20 days. A, B)** Response to TKIs in bone (**A**) and lung (**B**) tumors detected by bioluminescence, mean ± SEM, n=8-16/group.
